# Mechanisms involved in IL-15 superagonist enhancement of anti-PD-L1 therapy

**DOI:** 10.1186/s40425-019-0551-y

**Published:** 2019-03-21

**Authors:** Karin M. Knudson, Kristin C. Hicks, Sarah Alter, Jeffrey Schlom, Sofia R. Gameiro

**Affiliations:** 1 Laboratory of Tumor Immunology and Biology, Center for Cancer Research, National Cancer Institute, National Institutes of Health, Bethesda, MD USA; 2 Altor Bioscience, a NantWorks company, Miramar, FL USA

**Keywords:** PD-L1, ALT-803, N-803, Tumor microenvironment

## Abstract

**Background:**

Immunotherapy targeting PD-1/PD-L1 fails to induce clinical responses in most patients with solid cancers. N-803, formerly ALT-803, is an IL-15 superagonist mutant and dimeric IL-15RαSushi-Fc fusion protein complex that enhances CD8^+^ T and NK cell expansion and function and exhibits anti-tumor efficacy in preclinical models. Previous in vitro studies have shown that IL-15 increases PD-L1 expression, a negative regulator of CD8^+^ T and NK cell function. Most reported preclinical studies administered N-803 intraperitoneally not subcutaneously, the current clinical route of administration. N-803 is now being evaluated clinically in combination with PD-1/PD-L1 inhibitors. However, the mechanism of action has not been fully elucidated. Here, we examined the anti­tumor efficacy and immunomodulatory effects of combining N-803 with an anti-PD-L1 antibody in preclinical models of solid carcinomas refractory to anti-PD-L1 or N-803.

**Methods:**

Subcutaneous N-803 and an anti-PD-L1 monoclonal antibody were administered as monotherapy or in combination to 4T1 triple negative breast and MC38-CEA colon tumor-bearing mice. Anti-tumor efficacy was evaluated, and a comprehensive analysis of the immune-mediated effects of each therapy was performed on the primary tumor, lung as a site of metastasis, and spleen.

**Results:**

We demonstrate that N-803 treatment increased PD-L1 expression on immune cells in vivo, supporting the combination of N-803 and anti-PD-L1. N-803 plus anti-PD-L1 was well-tolerated, reduced 4T1 lung metastasis and MC38-CEA tumor burden, and increased survival as compared to N-803 and anti-PD-L1 monotherapies. Efficacy of the combination therapy was dependent on both CD8^+^ T and NK cells and was associated with increased numbers of these activated immune cells in the lung and spleen. Most alterations to NK and CD8^+^ T cell phenotype and number were driven by N-803. However, the addition of anti-PD-L1 to N-803 significantly enhanced CD8^+^ T cell effector function versus N-803 and anti-PD-L1 monotherapies, as indicated by increased Granzyme B and IFNγ production, at the site of metastasis and in the periphery. Increased CD8^+^ T cell effector function correlated with higher serum IFNγ levels, without related toxicities, and enhanced anti-tumor efficacy of the N-803 plus anti-PD-L1 combination versus either monotherapy.

**Conclusions:**

We provide novel insight into the mechanism of action of N-803 plus anti-PD-L1 combination and offer preclinical proof of concept supporting clinical use of N-803 in combination with checkpoint inhibitors, including for patients non- and/or minimally responsive to either monotherapy.

**Electronic supplementary material:**

The online version of this article (10.1186/s40425-019-0551-y) contains supplementary material, which is available to authorized users.

## Introduction

The gamma c (γc) cytokine interleukin (IL)-15 is a promising immunotherapy. IL-15 promotes CD8^+^ T and natural killer (NK) cell activation, proliferation, cytotoxicity, and survival [1]. N-803, previously known as ALT-803, is an IL-15 superagonist mutant complexed to a dimeric IL-15RαSushi-Fc fusion protein [2–4]. This fully humanized complex enhances IL-15 biological activity and stability in vivo [2–4] and promotes greater activation of CD8^+^ T cells and NK cells than recombinant IL-15 (rIL-15) with less toxicity [5–8]. N-803 was initially delivered intravenously (i.v.) with no observed dose limiting toxicities (DLTs); however, subcutaneous (s.c.) delivery of N-803 attenuated many adverse events, increased serum persistence, and expanded the number of circulating NK and CD8^+^ T cells as compared to systemic administration [7]. Consequentially, N-803 is now administered subcutaneously in the clinic. While preclinical studies with N-803 have demonstrated anti-tumor efficacy against multiple murine solid carcinomas, including breast [9], colon [5], and glioblastoma [10], these studies administered N-803 systemically via intraperitoneal (i.p.) injection. Pharmacokinetics, biodistribution, immune stimulation, and efficacy of s.c. versus i.v. administration were previously evaluated in a 5 T33 tumor model [11], but the efficacy of s.c.-administered N-803 on murine solid tumors and the mechanism of action have not been investigated.

In most murine solid carcinoma studies, N-803 monotherapy was not curative [5, 9, 10]. This may be due to immune suppression in the tumor microenvironment (TME), which includes the expression of checkpoint inhibitor molecules such as programmed cell death ligand-1 (PD-L1). Aberrant overexpression of PD-L1, which inhibits immune cell maturation, proliferation, and effector function [12, 13], is observed in numerous malignancies and is associated with poor clinical outcome [14, 15]. Antibodies blocking PD-L1 or programmed cell death-1 (PD-1) have increased patient survival in carcinomas with significant mutational burden such as melanoma [16, 17], lung [18, 19], and bladder cancers [20, 21]. However, most patients with solid carcinomas fail to respond to PD-1/PD-L1 blockade, including those with triple-negative (TN) breast and colon carcinomas [12]. Thus, there is an unmet need to develop combination therapies that improve clinical benefit for these patients.

One study recently demonstrated that rIL-15 induces PD-L1 expression in vitro on immune cells [22], providing further rationale for the combination of N-803 and PD-L1–targeted agents. The effect of N-803 on PD-L1 expression in vivo has not been reported. Preliminary studies of N-803 (i.p.) combination with anti-PD-1 (αPD-1) or anti-PD-L1 (αPD-L1) monoclonal antibodies have shown mixed results in murine solid carcinoma models [9, 10]. However, a recent Phase 1b clinical trial reported that the combination of N-803 with the αPD-1 antibody nivolumab in metastatic non-small cell lung cancer (NSCLC) patients was well-tolerated, and 29% of patients exhibited an objective response [23]. Of note, the addition of N-803 promoted objective responses in 3/11 (27%) of patients previously treated with PD-1 therapy [23], which suggests this agent may aid in overcoming acquired resistance to checkpoint blockade. A comprehensive analysis of the immune mechanisms of this combination has not been reported preclinically or clinically.

Here, for the first time, we describe the anti-tumor efficacy of subcutaneously administered IL-15 superagonist N-803 (previously known as ALT-803) in combination with an αPD-L1 monoclonal antibody in murine TN breast and colon carcinoma models. Furthermore, we report the immune-mediated mechanism of action of N-803 plus αPD-L1 combination therapy.

## Materials and methods

### Reagents

N-803 was generously provided by NantBioScience under a Cooperative Research and Development Agreement with the National Cancer Institute. αPD-L1 (10F.9G2), CD8 (2.43), and CD4 (GK1.5) depletion antibodies were from BioXcell. The NK depletion antibody (anti-asialo-GM1) was from Wako Chemicals.

### Mice

Six- to 10-week old female Balb/c and C57BL/6 mice transgenic for carcinoembryonic antigen (CEA) (designated C57BL/6-CEA) [24] were obtained from the NCI Frederick Cancer Research Facility and maintained under specific pathogen-free conditions in accordance with the Association for Assessment and Accreditation of Laboratory Animal Care (AAALAC) guidelines. All studies were approved by the NIH Intramural Animal Care and Use Committee (IACUC).

### Murine tumor cell lines and tumor studies

4T1 TN breast carcinoma and Yac-1 lymphoma cells were obtained from American Type Culture Collection (ATCC). MC38 colon carcinoma cells expressing human CEA (MC38-CEA) were generated as previously reported [25]. All cell lines were cultured according to providers’ instructions [25], determined as *mycoplasma* free by MycoAlert Mycoplasma Detection Kit (Lonza), and used at low passage number.

For anti-tumor studies, 4T1 tumor cells (5 × 10^4^, s.c.) were orthotopically implanted into the mammary fat pad of female Balb/c mice on day 0. In select studies, the primary tumor was surgically excised at day 15. MC38-CEA (5 × 10^5^, s.c.) tumor cells were implanted into the right flank of female C57BL/6-CEA mice. Tumors were measured biweekly using calipers, and volumes were determined as (length^2^ × width)/2. Mice were randomized based on tumor size and treatment initiated when tumors reached 50-100 mm^3^. Mice received three doses of 200 μg αPD-L1 i.p. (10 mg/kg), a clinically relevant dose [21], and/or two doses of N-803 s.c. at 1 μg [9]. Quantification of 4T1 lung metastasis was performed as previously described [26, 27].

### Depletion studies

CD4 or CD8 depletion antibodies (100 μg, i.p.) were administered on days 6, 7, and 8 post-tumor implant followed by once weekly. NK depletion antibody (25 μl in 100 μl PBS, i.p.) was administered on days 6 and 8 post-tumor implant, then every 3 days. Due to toxicity, depletions were terminated after day 19. Weekly depletion efficiency was determined in the blood (~ 50 μl) by flow cytometry. Percent reduction of CD8^+^ T, NK, or CD4^+^ T cells was determined versus undepleted N-803 + αPD-L1-treated mice (set to 0%).

### Isolation of immune cells

Homogenized and filtered spleens underwent ACK lysis. Tumors and lungs were digested using the gentleMACS Dissociator according to the manufacturer’s instructions (Miltenyi Biotec). Tumor-infiltrating lymphocytes (TILs) and lung-resident immune cells were enriched using a 44%/67% Percoll Plus (GE Healthcare) gradient. Cell counts were performed using 123count eBeads (ThermoFisher Scientific).

### Flow cytometry and antibodies

Antibody labeling of cells for flow cytometry (1–10 × 10^6^ immune cells) was performed using the BD Cytofix/Cytoperm Kit (BD Biosciences) according to the manufacturer’s instructions. Antibodies (Additional file 1: Table S1) and matched isotypes were obtained from the listed manufacturers. Live/Dead Fixable Dead Cell Stain was from Invitrogen. Flow cytometry (≥1 × 10^5^ events) was performed on a BD FACSVerse or LSRFortessa flow cytometer (Becton Dickinson) and analyzed with FlowJo FACS Analysis Software v9.9.6 (Treestar). Cell populations were identified as listed (Additional file 1: Table S2). Expression of phenotypic proteins was determined by subtracting the respective isotype, set between 1 and 5% of the population.

### Discrimination of immune cells in lung parenchyma versus vasculature

To discriminate the presence of lung parenchymal versus intravascular immune cells, mice were injected with anti-CD45.2 (3 μg, i.v.) as previously described [28]. See Additional file 1: Figure S1.

### CD8^+^ T cell and NK cell restimulation

Isolated spleen, lung, and tumor immune cells were stimulated for 4 h in the presence of 2 μg/ml GolgiPlug (BD Biosciences) with nothing, for the CD8^+^ T cell restimulation 1 μg/ml αCD3 (2C11, BD Biosciences) + 1 μg/ml αCD28 (37.51, BD Biosciences), or for the NK cell restimulation 50 ng/ml PMA (Sigma) + 500 ng/ml ionomycin (Sigma). Frequencies of stimulated IFNγ^+^ and/or TNFα^+^ cells were calculated by subtracting the non-stimulated controls.

### NK cell cytotoxicity assay

Purified splenic NK cells (NK Cell Isolation Kit, Miltenyi Biotec) were co-cultured for 18 h with ^111^In-labeled Yac-1 target cells at a 100:1 effector-to-target ratio. Supernatant ^111^In radioactivity was quantified (WIZARD^2^ γ counter, PerkinElmer), and percent lysis was determined as previously described [9].

### Detection and quantification of serum cytokines

Serum cytokines were quantified using the V-PLEX Proinflammatory Panel I Mouse Kit and MESO QuickPlex SQ 120 (Meso Scale Diagnostics, LLC). Limits of detection were: IFNγ: 0.04 pg/ml, IL-6: 0.61 pg/ml, IL-10: 0.95 pg/ml, TNFα: 0.13 pg/ml.

### Statistics

Statistical analyses were performed in Prism 7.0a (GraphPad Software). Unless otherwise stated, data presented in bar graphs or scatter plots were analyzed using one-way ANOVA with Tukey’s multiple comparisons. Two-way ordinary ANOVA was used to analyze tumor growth curves. Survival was analyzed using Log-rank (Mantel-Cox) test. Outliers were identified using ROUT test. Statistical significance was set at *p* < 0 .05. *p < 0.05, **p < 0.01, ***p < 0.001.

## Results

### N-803 increases PD-L1 expression on hematopoietic cells

**Fig. 1:**
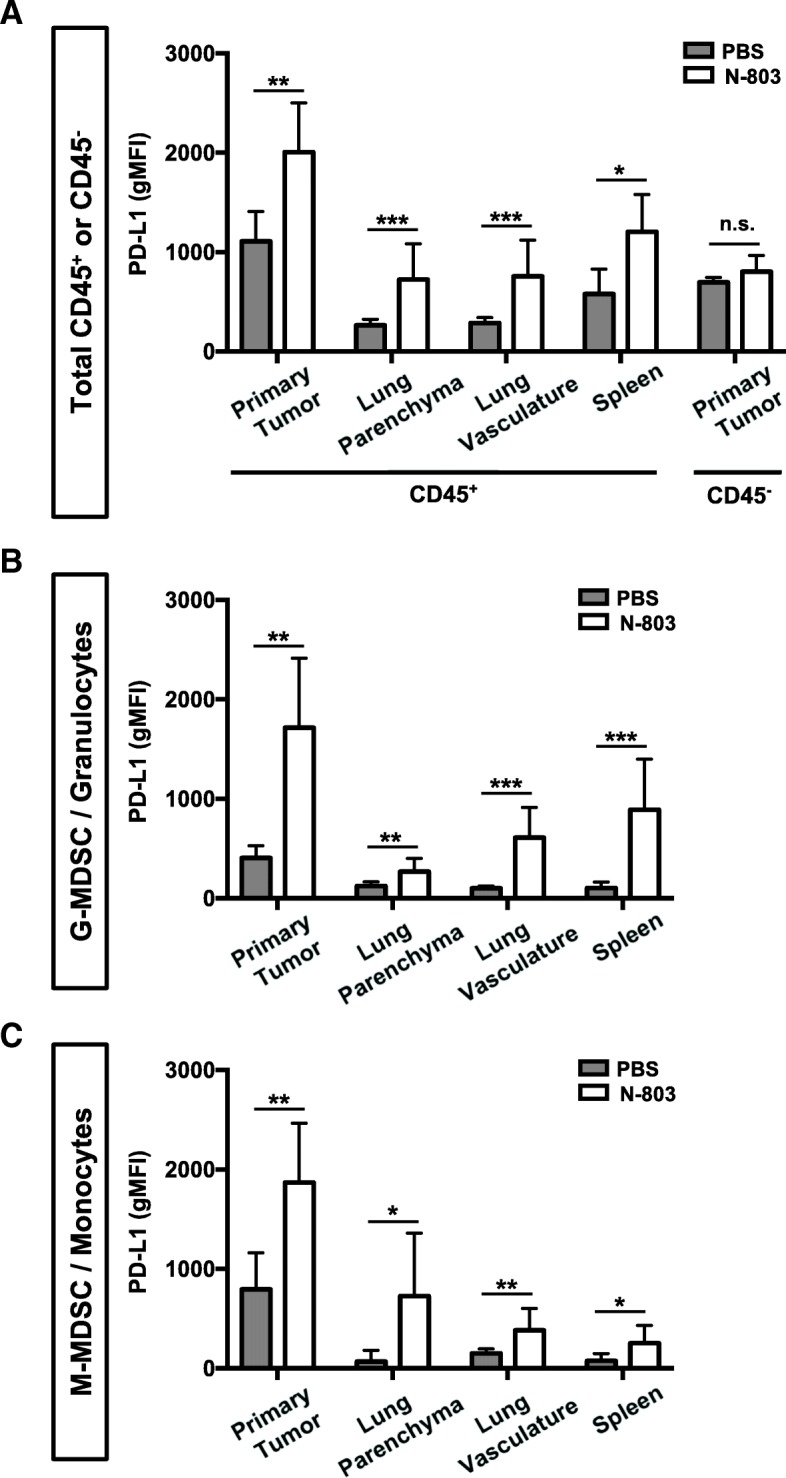
N-803 treatment increases PD-L1 expression on total CD45^+^ cells and MDSC populations in the primary tumor, lung, and spleen. 5 × 10^4^ 4T1 tumor cells were orthotopically implanted into female Balb/c mice. When tumor volumes reached ~50mm^3^, mice were treated at days 9 and 13 with 1 μg N-803 (s.c.). Twenty-four hours after the last treatment, PD-L1 expression (geometric mean fluorescence intensity (gMFI)) in the primary tumor, lung parenchyma and vasculature, and spleen was determined by flow cytometry on total CD45^+^ or CD45^−^ cells (**a**), G-MDSC/Granulocytes (**b**), or M-MDSC/Monocytes (**c**). Graphs show mean ± SD. Data combined from 2 independent experiments, *n* = 5 mice/group per experiment

N-803 reduces tumor burden in multiple murine solid carcinoma models. However, in many cases, it was not curative [5, 9]. It has been shown that IL-15 increases PD-L1 expression on immune cells in vitro [22], but the effect of rIL-15 or N-803 on PD-L1 expression in vivo has not been reported. Thus, it is possible that N-803 enhances PD-L1 expression in tumor-bearing mice, reducing the efficacy of N-803 monotherapy. Indeed, s.c.-delivered N-803 enhanced PD-L1 expression on CD45^+^ hematopoietic cells in the primary tumor, lung parenchyma and vasculature, and spleen of 4T1 TN breast tumor-bearing mice (Fig. 1a). In addition, granulocytic myeloid derived suppressor cell (G-MDSC)/granulocyte and monocytic MDSC (M-MDSC)/monocyte populations had increased expression of PD-L1 (Fig. 1b,c). PD-L1 expression on non-hematopoietic, tumor-associated CD45^−^ cells did not change (Fig. 1a). These results demonstrate that N-803 increases PD-L1 expression on immune cells in vivo and provide the rationale for combining N-803 with a PD-L1–targeted antibody for the treatment of solid carcinomas.

### N-803 + αPD-L1 combination decreases tumor burden and improves overall survival in murine breast and colon carcinoma models

Given the increase in PD-L1 expression upon N-803 treatment, we examined the combination of N-803 with an αPD-L1 monoclonal antibody for anti-tumor efficacy. Successful combination of these agents has not been reported [9], and previous studies using N-803 with checkpoint blockade did not use subcutaneous delivery of N-803, the route of administration used in the clinic [7, 8, 23].

First, we examined the efficacy of N-803 plus αPD-L1 (N-803 + αPD-L1) combination therapy in mice bearing 4T1 TN breast tumors, which are highly metastatic [29] and have previously demonstrated resistance to αPD-L1 monotherapy [30]. Initial studies determined that 1 μg s.c. N-803 in combination with 200 μg (10 mg/kg) αPD-L1, a clinically relevant dose [21], promoted greater reduction in lung tumor burden than higher doses of N-803 (Additional file 1: Figure S2A-C). Furthermore, significant reduction in body weight was observed after treatment with higher doses of N-803 (Additional file 1: Figure S2D-E). Thus, all studies were performed using 1 μg N-803 s.c. plus 200 μg αPD-L1 i.p.

**Fig. 2:**
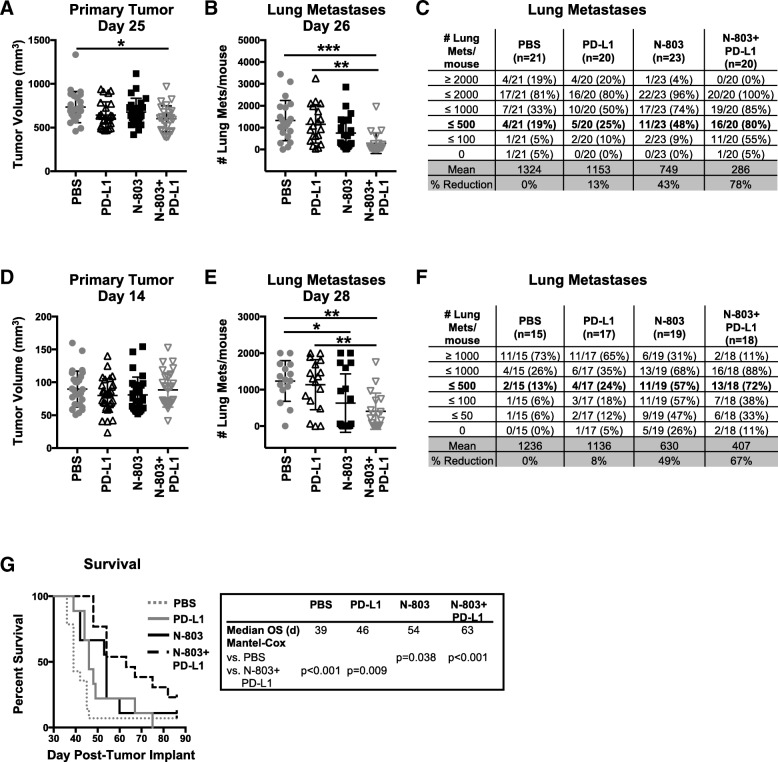
Combination of N-803 and αPD-L1 decreases 4T1 lung metastasis and improves survival. **a**-**c** Mice were implanted with 4T1 tumors as in Fig. 1 and treated on days 8 and 14 with 1 μg N-803 (s.c.) and/or 200 μg αPD-L1 (i.p.) on days 8, 11, and 14. Graphs of tumor volumes of individual animals at day 25 post-tumor implant in (**a**) and number of lung metastases in individual mice 26 days after tumor implant (**b**) show mean ± SD. **c** Table denotes the distribution of number of lung metastases and % reduction in mean versus PBS. Data are representative of 2 independent experiments, *n* = 20–23 mice. **d**-**g** Mice were implanted with 4T1 tumors as in Fig. 1 and treated at days 9 and 13 with N-803 and/or αPD-L1 on days 9, 11, and 13. The primary tumor was surgically resected at day 15. Graphs of tumor volumes of individual animals at day 14 post-tumor implant (**d**) and number of lung metastases in individual mice at day 28 (**e**) show mean ± SD. **f** Table denotes the distribution of number of lung metastases per mouse and % reduction in mean versus PBS. Data are representative of 2 independent experiments, *n* = 15–19 mice. **g** Survival curves (inset: mOS) show % survival. Data are representative of 2 independent experiments, *n* = 13–19 mice

In the adjuvant setting, without resection of the primary tumor, N-803 + αPD-L1 reduced primary 4T1 tumor growth (Fig. 2a) and decreased the number of lung metastases by 78% compared to phosphate buffered saline (PBS) treatment (Fig. 2b,c). Neither αPD-L1 nor N-803 monotherapy significantly reduced primary or lung tumor burden compared to PBS treatment (Fig. 2a-c). Combination therapy further reduced lung metastasis burden by 75 and 62% compared to αPD-L1 and N-803 monotherapies, respectively (Fig. 2c).

Most breast cancer patients have their primary lesion surgically removed. To mimic the clinical regimen, we surgically resected primary 4T1 tumors 15 days after implant. None of the treatments reduced primary tumor growth prior to resection (Fig. 2d). αPD-L1 monotherapy did not affect 4T1 lung metastasis, but N-803 monotherapy significantly reduced lung tumor burden by 49% as compared to PBS treatment (Fig. 2e,f). The addition of αPD-L1 to N-803 improved this response and promoted a 67% reduction in lung metastasis burden versus PBS (Fig. 2e,f). The observed decrease in lung metastasis with N-803 monotherapy and N-803 + αPD-L1 correlated with significant increases in median overall survival (mOS) as compared to PBS- and αPD-L1-treated mice (Fig. 2g). N-803 and N-803 + αPD-L1 increased survival by 38 and 62% versus PBS treatment, respectively (Fig. 2g).

We also examined the anti-tumor efficacy of N-803 + αPD-L1 therapy in the aggressive murine MC38-CEA colon carcinoma model. N-803 + αPD-L1 significantly reduced tumor burden compared to PBS treatment, with 43% of mice cured (Additional file 1: Figure S3A,B) and an 83% increase in mOS (Additional file 1: Figure S3C)*.* N-803 did not reduce tumor burden in this model. However, αPD-L1 monotherapy cured 12.5% of mice but did not significantly improve mOS (Additional file 1: Figure S3A-C). αPD-L1 and N-803 + αPD-L1 therapies promoted long-term immunity as MC38-CEA rechallenge of cured mice did not induce palpable tumors (Additional file 1: Figure S3D,E).

All together, these results demonstrate for the first time that subcutaneously delivered N-803 in combination with αPD-L1 decreases tumor burden and improves overall survival in multiple murine solid carcinoma models. Importantly, N-803 + αPD-L1 combination increases anti-tumor efficacy versus both N-803 and αPD-L1 monotherapies.

### Anti-tumor efficacy of N-803 + αPD-L1 therapy is dependent on NK and CD8^+^ T cells

**Fig. 3:**
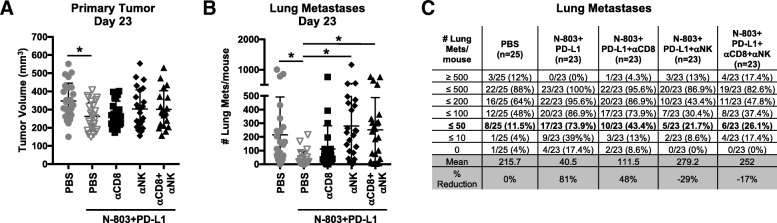
CD8^+^ T cells and NK cells contribute to the anti-tumor efficacy of N-803 + αPD-L1 combination. Mice were implanted with 4T1 tumors as in Fig. 1 and treated on days 13 and 17 with N-803 and αPD-L1 on days 13, 15, and 17. CD8-expressing cells and NK cells were depleted on days 10, 11, 12, 16, and 19 using 100 μg anti-CD8 and/or 25 μl anti-asialo-GM1 (i.p.). Graphs show tumor volumes of individual mice (**a**) or number of lung metastases in individual mice at day 23 post-tumor implant (**b**) as mean ± SD. **c** Table denotes the distribution of the number of lung metastases per mouse and % reduction in mean versus PBS. Data combined from 2 independent experiments, *n* = 23–25 mice/group total

To begin elucidating the mechanism by which N-803 + αPD-L1 mediated anti-tumor efficacy, we first determined the immune cells required to reduce tumor burden in 4T1 tumor-bearing mice. As CD8^+^ T and NK cells were necessary for the anti-tumor efficacy of N-803 monotherapy in the 4T1 model [9], we performed CD8^+^ T cell and NK cell depletions in N-803 + αPD-L1–treated mice. Vascular CD8^+^ T cells and NK cells were depleted by ~ 98% and ~ 90%, respectively, over the first 3 weeks of the experiment (Additional file 1: Figure S4A-C). N-803 + αPD-L1 significantly reduced primary tumor growth and lung metastasis versus PBS treatment (Fig. 3a-c). While CD8^+^ T cell depletion did not significantly increase the number of lung metastases versus N-803 + αPD-L1 therapy, it did increase mean lung tumor burden by 175% (Fig. 3b,c). Furthermore, there was no significant difference in the number of lung metastases in CD8-depleted mice compared to PBS treatment (Fig. 3b,c). The NK cell depletion, on the other hand, significantly increased lung tumor burden by 589% versus N-803 + αPD-L1 therapy (Fig. 3b,c). Depletion of both CD8^+^ T cells and NK cells mimicked the results seen with NK cell depletion (Fig. 3a-c). Of note, 99% depletion of CD4^+^ T cells had no effect on the anti-tumor efficacy of N-803 + αPD-L1 (Additional file 1: Figure S4D-G). These results indicate NK cells, and to a lesser extent CD8^+^ T cells, are required for the anti-tumor efficacy of N-803 + αPD-L1 therapy in 4T1 tumor-bearing mice.

In order to confirm the requirement of CD8^+^ T cells and NK cells for responsiveness to N-803 + αPD-L1 therapy, we also performed CD8^+^ T cell and NK cell depletions in MC38-CEA tumor-bearing mice. N-803 + αPD-L1 reduced primary tumor burden (Additional file 1: Figure S5A,B) and increased mOS by 33% versus PBS treatment (Additional file 1: Figure S5C). Depletion of CD8^+^ T cells, NK cells, and both CD8^+^ T cells and NK cells completely abrogated the anti-tumor efficacy of N-803 + αPD-L1 (Additional file 1: Figure S5A,B). All of the depletion strategies similarly reduced mOS by 45 and 9% versus N-803 + αPD-L1 and PBS treatment, respectively (Additional file 1: Figure S5C). Thus, CD8^+^ T cells and NK cells are equally required for the efficacy of N-803 + αPD-L1 in MC38-CEA tumor-bearing mice.

### N-803 + αPD-L1 treatment decreases T_reg_ and G-MDSC numbers in the lung

To further elucidate the mechanism of N-803 + αPD-L1–induced anti-tumor efficacy, we performed a comprehensive characterization of immune cell populations in the primary tumor, in the lung as a site of metastasis, and in the peripheral immune compartment in the spleen of 4T1 tumor-bearing mice 24 h after the last N-803 and/or αPD-L1 treatment. The effect of N-803 + αPD-L1 combination on the immune system in murine solid carcinoma models has not been previously reported.

While we were particularly interested in NK and CD8^+^ T cells, given the results of the depletion studies, we first investigated the effect of N-803 + αPD-L1 therapy on regulatory T cell (T_reg_) and MDSC populations, as these immunosuppressive cells play important roles in the progression of 4T1 tumors [31, 32]. T_reg_ numbers were significantly decreased in the lung vasculature, but not in other immune compartments, with all treatments (Additional file 1: Figure S6). In addition, N-803 + αPD-L1 treatment decreased the number of G-MDSC/granulocytes in the lung parenchyma and vasculature (Additional file 1: Figure S7A). αPD-L1 and N-803 also reduced G-MDSC/granulocytes in the lung parenchyma or vasculature, respectively (Additional file 1: Figure S7A). There was no effect on the number or frequency of the M-MDSC/monocyte population (Additional file 1: Figure S7B). Thus, N-803 + αPD-L1 combination decreases immunosuppressive T_reg_ and G-MDSC/granulocyte populations in the lung.

### N-803 and N-803 + αPD-L1 treatments promote the development of an activated NK cell phenotype in the lung and spleen and increase NK cell function

**Fig. 4:**
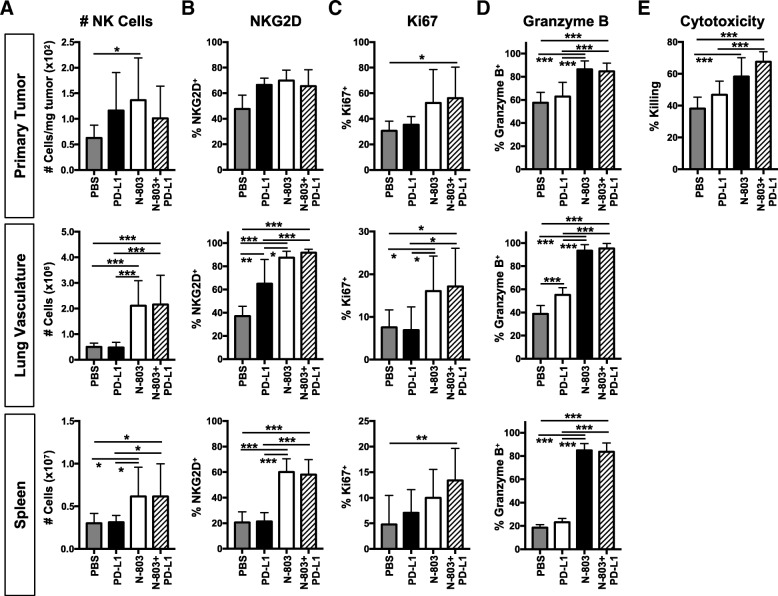
N-803 monotherapy and N-803 + αPD-L1 combination promote an activated NK cell phenotype and increase function. Mice were implanted with 4T1 tumors as in Fig. 1 and treated on days 9 and 13 with N-803 and/or αPD-L1 on days 9, 11, and 13. **a**-**d** NK cells were examined by flow cytometry in the primary tumor, lung vasculature, and spleen 24 h after the last treatment. Graphs show NK cell number (**a**) and frequencies of NKG2D^+^ (**b**), Ki67^+^ (**c**), and Granzyme B^+^ (**d**) NK cells. **e** Purified splenic NK cells were co-cultured with ^111^In-labeled Yac-1 target cells at a 100:1 effector-to-target (E:T) ratio for 18 h. ^111^In release was measured to determine cytotoxic function. All graphs show mean ± SD. Data combined from 2 to 3 independent experiments, *n* = 4–5 mice/group per experiment

Given the necessity of NK cells for N-803 + αPD-L1 anti-tumor efficacy, we examined the effect of combination therapy on NK cell phenotype. Furthermore, while N-803 is known to greatly increase the number and activation of NK cells in the tumor and spleen of tumor-bearing mice [9–11], the effect of subcutaneous N-803 plus checkpoint blockade on NK cell phenotype and function has not been reported. In the primary tumor, N-803 + αPD-L1 did not affect NK cell number (Fig. 4a), frequency (Additional file 1: Figure S8A), or expression of activation-associated receptors NKG2D (Fig. 4b) or NKp46 (Additional file 1: Figure S8B). There was, however, a significant increase in the proportion of Ki67^+^ NK cells (Fig. 4c), indicating an increased proliferative potential.

Given the significant reduction in lung tumor burden with N-803 + αPD-L1 (Fig. 2), NK cell populations in the lung were of particular interest. We could not detect NK cells in the lung parenchyma due to low cell number, but we did examine NK cells in the lung vasculature. N-803 + αPD-L1 greatly increased the number (Fig. 4a) and frequency (Additional file 1: Figure S8A) of vascular NK cells in the lung versus PBS and αPD-L1. The proportion of NK cells expressing NKG2D (Fig. 4b) and Ki67 (Fig. 4c) and expression of NKp46 (Additional file 1: Figure S8B) were also significantly enhanced with N-803 + αPD-L1 versus PBS treatment. N-803 + αPD-L1–induced changes to splenic NK cell populations were similar to those observed in the lung vasculature (Fig. 4a-c and Additional file 1: Figure S8A,B). The increased cell numbers and expression of activation-associated proteins with N-803 + αPD-L1 treatment appeared to be driven by N-803, as similar increases were observed with N-803, but not αPD-L1, monotherapy (Fig. 4a-c and Additional file 1: Figure S8A,B).

The changes to NK cell phenotype with N-803 + αPD-L1 combination suggested that NK cell function would be markedly enhanced. As expected, N-803 + αPD-L1 treatment significantly increased the population of NK cells expressing the cytolytic molecule Granzyme B in the primary tumor, lung vasculature, and spleen versus PBS and αPD-L1 treatments (Fig. 4d and Additional file 1: Figure S8C). Increased Granzyme B expression correlated with a profound enhancement of NK cell killing of Yac-1 target cells with N-803 + αPD-L1 versus PBS and αPD-L1 treatments (Fig. 4e). In addition, a greater proportion of splenic and lung vascular NK cells produced IFNγ, and the amount of IFNγ produced on a per cell basis increased with N-803 + αPD-L1 therapy (Additional file 1: Figure S8D). Again, similar results were seen with N-803 and N-803 + αPDL1 treatments (Fig. 4d,e and Additional file 1: Figure S8C,D). Together, these results suggest that N-803 + αPD-L1 therapy supports anti-tumor efficacy through enhanced NK cell activation and function primarily in the lung and spleen, and this effect is mainly driven by N-803.

### N-803 + αPD-L1 therapy induces an activated CD8^+^ T cell phenotype in the lung and spleen and enhances effector function

**Fig. 5:**
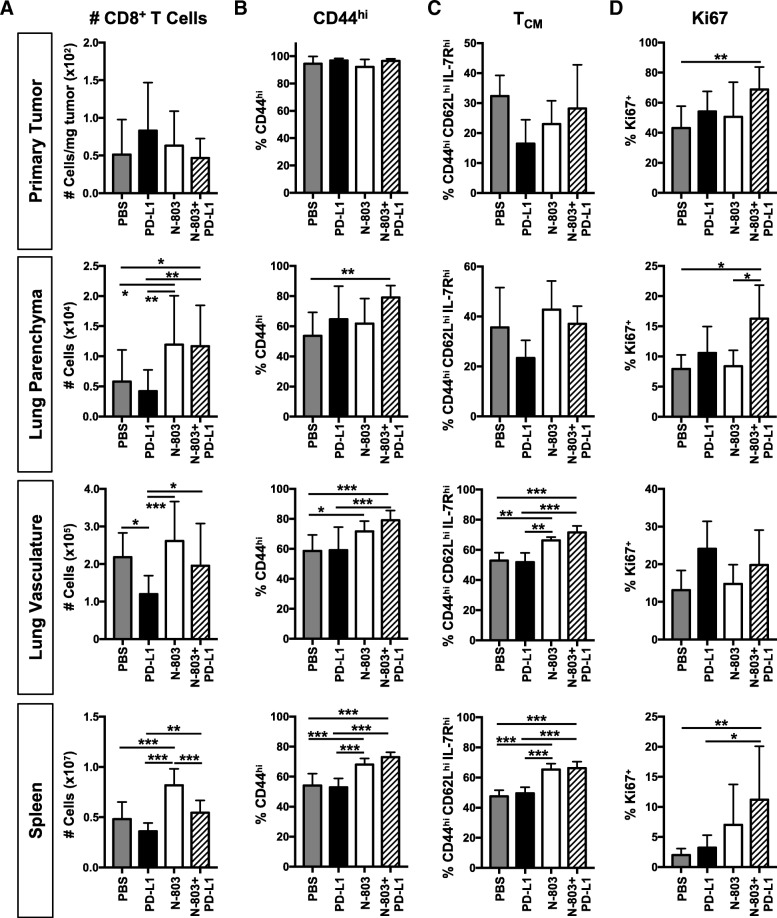
Combination of N-803 + αPD-L1 induces an activated CD8^+^ T cell phenotype. Mice were implanted with 4T1 tumors as in Fig. 1 and treated on days 9 and 13 with N-803 and/or αPD-L1 on days 9, 11, and 13. CD8^+^ T cells were examined by flow cytometry in the primary tumor, lung parenchyma and vasculature, and spleen 24 h after the last treatment. Graphs of CD8^+^ T cell number (**a**), frequency of CD44^hi^ (**b**), CD44^hi^CD62L^hi^ T_CM_ (**c**), and Ki67^+^ CD44^hi^ (**d**) CD8^+^ T cells show mean ± SD. Data combined from 2 to 3 independent experiments, *n* = 5 mice/group per experiment

The increased anti-tumor efficacy of N-803 + αPD-L1 versus N-803 monotherapy could not be fully explained by differences in NK cell phenotype and function, as both treatments induced similar changes to these cells. While CD8^+^ T cells contributed less to the reduction of 4T1 lung metastasis by N-803 + αPD-L1 than NK cells, they did partially support this anti-tumor efficacy (Fig. 3). It is known that both N-803 and αPD-L1 monotherapies enhance CD8^+^ T cell proliferation, activation, and function [9–12], but the effect of combining N-803 plus checkpoint blockade on CD8^+^ T cells has not been elucidated. Similar to NK cells, there were no significant differences in CD8^+^ T cell number (Fig. 5a) or phenotype (Fig. 5b-d) in the primary tumor with treatment, other than an increase in the frequency of Ki67^+^ CD8^+^ T cells (Fig. 5d), indicating an increased proliferative potential. In the lung, N-803 + αPD-L1 therapy enhanced lung parenchymal-resident CD8^+^ T cell number (Fig. 5a) and the proportion of activated CD44^hi^ (Fig. 5b) and Ki67-expressing (Fig. 5d) cells. In the lung vasculature, there was also a significant enrichment of the population of activated CD44^hi^ CD8^+^ T cells (Fig. 5b), specifically of a central memory (T_CM_) phenotype (Fig. 5c), after N-803 + αPD-L1 versus PBS treatment. N-803 + αPD-L1 therapy also greatly altered the splenic CD8^+^ T cell population. While N-803 + αPD-L1 did not affect CD8^+^ T cell number (Fig. 5a) versus PBS treatment, combination therapy augmented the frequency of Ki67^+^ (Fig. 5d) and activated CD44^hi^ CD8^+^ T cells (Fig. 5b), in particular those with a T_CM_ phenotype (Fig. 5c). Similar to NK cells, the changes in CD8^+^ T cell number and phenotype, with the exception of Ki67, appeared dependent on N-803, but not αPD-L1, monotherapy (Fig. 5).

Sustained, elevated levels of PD-1 on T cells can correlate with the development of CD8^+^ T cell exhaustion [12]. Both IL-15 treatment and PD-L1 blockade have been shown to reduce T cell exhaustion [1, 12], but the effect of N-803 + αPD-L1 therapy on this process has not been described. We examined the expression of PD-1 on CD8^+^ T cells after treatment. One day after the final treatment, at day 14, PD-1 expression was low on CD8^+^ T cells (Additional file 1: Figure S9). By day 21 post-tumor implant, CD8^+^ T cells expressed significantly less PD-1 after N-803 + αPD-L1 than PBS treatment (Additional file 1: Figure S9), suggesting they may be less exhausted. Importantly, this reduction was only seen with N-803 + αPD-L1 and not αPD-L1 or N-803 monotherapy (Additional file 1: Figure S9).

**Fig. 6:**
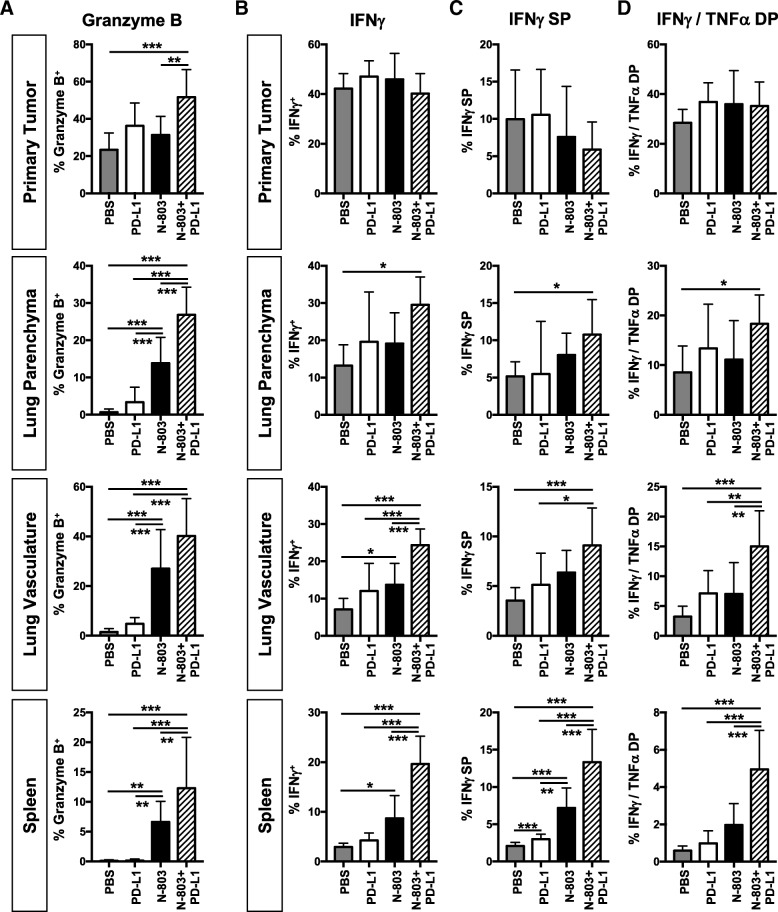
Combination of N-803 + αPD-L1 increases Granzyme B and effector cytokine production by CD8^+^ T cells. Mice were implanted with 4T1 tumors as in Fig. 1 and treated on days 12 and 16 with N-803 and/or αPD-L1 on days 12, 14, and 16. CD8^+^ T cell effector molecule and cytokine production were examined by flow cytometry in the primary tumor, lung parenchyma and vasculature, and spleen 24 h after last treatment. **a** Graphs show frequency of Granzyme B^+^ CD44^hi^ CD8^+^ T cells. **b**-**d** Immune cells were stimulated with 1 μg/ml αCD3 + 1 μg/ml αCD28 for 4 h. Graphs show frequency of total IFNγ^+^ CD44^hi^ CD8^+^ T cells (**b**) and frequency of IFNγ-single producing (SP) (**c**) or IFNγ/TNFα-double producing (DP) (**d**) CD44^hi^ CD8^+^ T cells. All graphs show mean ± SD. Data combined from 2 independent experiments, *n* = 5 mice/group per experiment

The development of a more active CD8^+^ T cell phenotype with N-803 + αPD-L1 treatment suggested that CD8^+^ T cell effector function would also be improved. After N-803 + αPD-L1 treatment, CD8^+^ T cells in the lung and spleen expressed more Granzyme B than all other treatment groups, including N-803 monotherapy (Fig. 6a and Additional file 1: Figure S10A). CD8^+^ T cells also expressed more Granzyme B in the primary tumor with N-803 + αPD-L1 treatment versus PBS and N-803 treatments (Fig. 6a and Additional file 1: Figure S10A). In addition, combination therapy significantly increased the proportion of all IFNγ-producing CD8^+^ T cells versus PBS treatment in the lung parenchyma and versus all other treatments in the lung vasculature and spleen (Fig. 6b). IFNγ production on a per cell basis was not altered with treatment (Additional file 1: Figure S10B). While the proportion of all TNFα-producing CD8^+^ T cells did not increase with N-803 + αPD-L1 treatment except in the spleen, there was a minor increase in TNFα production on a per cell basis as compared to PBS treatment in the lung (Additional file 1: Figure S10C). Together, the increased frequency of IFNγ-producing cells with N-803 + αPD-L1 therapy translated into a greater proportion of CD8^+^ T cells that were IFNγ single producers (IFNγ^+^TNFα^−^) (Fig. 6c) and IFNγ/TNFα (IFNγ^+^TNFα^+^) double producers (Fig. 6d).

Together, these results suggest that N-803 + αPD-L1 therapy improves CD8^+^ T cell activation and expansion in the peripheral immune compartments, and that this effect on CD8^+^ T cell phenotype is mainly driven by N-803. However, maximal enhancement of CD8^+^ T cell function requires N-803 and αPD-L1 in combination, as N-803 + αPD-L1 treatment increases effector molecule and cytokine production over both N-803 and αPD-L1 monotherapies at the tumor sites and in the periphery.

### N-803 + αPD-L1 treatment induces high levels of serum immunostimulatory cytokines IFNγ and TNFα in the absence of toxicity

**Fig. 7:**
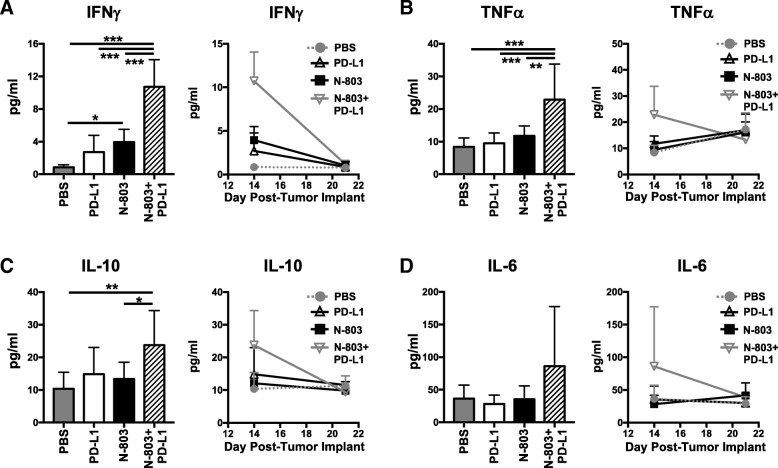
N-803 + αPD-L1 combination promotes the generation of an immunostimulatory milieu in the serum. Mice were implanted with 4T1 tumors as in Fig. 1 and treated on days 9 and 13 with N-803 and/or αPD-L1 on days 9, 11, and 13. Serum was obtained at days 14 and 21 post-tumor implant and analyzed for level of IFNγ (**a**), TNFα (**b**), IL-10 (**c**), and IL-6 (**d**). Level of serum cytokines from individual animals at day 14 (left panels) and curves showing kinetics of serum cytokine levels (right panels) show mean ± SD. Data are combined from 2 experiments, *n* = 4–5 mice/group per experiment

Together, the aforementioned results suggest that N-803 + αPD-L1 therapy would induce significantly higher global levels of immunostimulatory cytokines IFNγ and TNFα, driven by CD8^+^ T and NK cell production, as compared to αPD-L1 and N-803 monotherapies. Along with the proinflammatory cytokine IL-6, sustained high levels of IFNγ and TNFα can induce significant clinical toxicities, as was seen in early rIL-15 clinical trials [6–8]. Thus, we examined the kinetics of global serum cytokine levels. Twenty-four hours after the last treatment, N-803 + αPD-L1 therapy induced significantly higher levels of serum IFNγ (Fig. 7a) and TNFα (Fig. 7b) than all other treatments. The anti-inflammatory cytokine IL-10 (Fig. 7c) was also markedly enhanced versus PBS and N-803 therapy. The increased IFNγ, TNFα, and IL-10 levels were transient, as they returned to PBS-treated concentrations by day 21 or 7 days after treatment (Fig. 7a-c). IL-6 was not significantly increased in the serum with N-803 + αPD-L1 treatment (Fig. 7d). Importantly, even with the highly elevated levels of IFNγ with N-803 + αPD-L1 treatment, the combination was well-tolerated. At 1 μg N-803 plus 200 μg αPD-L1, there was no significant loss of body weight observed when compared to PBS treatment (Additional file 1: Figure S2D-E). In all, these results support that N-803 + αPD-L1 combination is uniquely able to induce a transient, immunostimulatory environment that promotes significant anti-tumor efficacy without inducing significant toxicity.

## Discussion

Cytokine therapy and immune checkpoint blockade are two major immunotherapy modalities in oncology. In the studies presented here, we combined the IL-15/IL-15RαSushi-Fc superagonist N-803 with a PD-L1-targeted antibody. To our knowledge, these studies are the first to evaluate the anti-tumor efficacy of N-803 administered subcutaneously, the route used in the clinic [7, 8, 23], as a monotherapy or in combination with checkpoint inhibition in murine models of solid carcinomas. N-803 + αPD-L1 treatment was well-tolerated, elicited significant anti-tumor efficacy, and improved survival in the 4T1 TN breast and MC38-CEA colon carcinoma models. This is in contrast to monotherapy-treated mice, where each model responded to different components of the combination therapy in isolation. While 4T1 lung metastasis was refractory to αPD-L1 therapy, it was partially responsive to N-803. MC38-CEA tumors responded in the opposite fashion. These results uniquely demonstrate that N-803 + αPD-L1 combination elicits broader anti-tumor efficacy as compared to either monotherapy. This could be beneficial in the clinic where tumor type can determine resistance to immunotherapy and many patients with solid carcinomas have an innate or acquired resistance to checkpoint blockade [33, 34]. As reported here using the 4T1 model, the addition of N-803 to αPD-L1 therapy may convert αPD-L1 poor or non-responding patients into responders. This is supported by a recent clinical study (NCT02523469) in which 91% of PD-1 treatment-resistant metastatic NSCLC patients achieved disease control, with 27% partial responses and 64% stable disease after concurrent treatment with N-803 and the αPD-1 antibody nivolumab [23]. It is possible that similar results will be seen in patients treated with the combination of N-803 and αPD-L1. Unfortunately, direct examination of N-803-mediated reversion of acquired αPD-L1 resistance is not feasible using these aggressive murine tumor models. The duration of these experiments is ethically limited due to tumor growth to approximately 4 weeks, preventing long-term experiments where mice could be dosed sequentially with αPD-L1 followed by N-803. In addition, repeated dosing with the αPD-L1 antibody (rat) or N-803 (human) can generate anti-rat and anti-human immune responses, respectively, reducing the efficacy of these treatments over time with potential severe toxicity. It has been reported that repeated dosing of 4T1 tumor-bearing mice with αPD-L1 antibodies induces fatal hypersensitivity [35].

While the mechanism of action of N-803 monotherapy has been investigated in multiple preclinical murine models and in patients [4, 5, 7, 9, 10], the studies presented here are the first to report the immune mechanism by which N-803 + αPD-L1 combination mediates anti-tumor efficacy. Furthermore, these studies are the first to comprehensively interrogate and compare the immune response in multiple immune compartments, including the primary tumor, a site of metastasis, and in the periphery. Similar to other N-803 monotherapy studies [4, 9], efficacy of N-803 + αPD-L1 was dependent on NK and CD8^+^ T cells, but the relative contributions of these two immune cell populations was dependent on the tumor model and tumor site analyzed. In the 4T1 TN breast carcinoma model, depletion of NK cells abrogated the reduction in lung tumor burden by N-803 + αPD-L1, while loss of CD8^+^ T cells increased the mean number of lung metastases by 175%. This suggests that for control of lung metastases, NK cells can compensate for the loss of CD8^+^ T cells, but CD8^+^ T cells cannot compensate for the loss of NK cells. NK cells have previously been shown to be important for immunosurveillance of the lung [36], a unique immunological environment highly dependent on innate immune surveillance due to its constant expose to exogenous particles, and to contribute to the detection and elimination of tumor cells during the initial immune response in the lung [37, 38]. By contrast, both CD8^+^ T cells and NK cells highly contributed to the anti-tumor efficacy of N-803 + αPD-L1 in MC38-CEA colon carcinoma model, with loss of either immune population increasing primary tumor growth. Thus, a therapy such as N-803 + αPD-L1, which targets both CD8^+^ T and NK cells, may support broader tumor control than a therapy that affects only one of these immune cell populations.

Here, we demonstrated that both CD8^+^ T cells and NK cells were expanded in peripheral immune sites and displayed enhanced expression of activation-associated proteins with N-803 + αPD-L1. These results are similar to those observed in the Phase I clinical trial with the combination of nivolumab plus N-803. In the peripheral blood of treated patients, NK cells more than doubled within 7 days post-treatment [23]. While CD8^+^ T cells did not expand to the same extent, both CD8^+^ T cells and NK cells expressed 2- and 3-fold higher levels of Ki67 after 4 days, respectively [23]. Likewise, we observed a greater expansion of NK cells than CD8^+^ T cells in the lung vasculature and spleen 2 days after the final treatment with N-803 and N-803 + αPD-L1, as well as significant upregulation of Ki67 in both immune populations in the spleen. While the effects on both CD8^+^ T cell and NK cell phenotype were highly dependent on the N-803 component, with minimal differences observed between the immune compartments in N-803- and N-803 + αPD-L1–treated mice, CD8^+^ T cell function was uniquely altered with combination therapy. Production of Granzyme B and IFNγ by CD8^+^ T cells was significantly improved with combination therapy versus all other treatments, including N-803 monotherapy, in the lung. Additionally, CD8^+^ T cell production of IFNγ was similarly enhanced in primary tumor. Thus, we hypothesize that increased CD8^+^ T cell function is at least partially responsible for the enhanced anti-tumor efficacy of N-803 + αPD-L1 versus N-803 monotherapy. Investigation of biomarkers of responsiveness to this combination therapy is of interest, including the role of tumor MHC Class I and PD-L1 level, capacity to mount a systemic IFNγ response, expansion of neoantigen-reactive T cells, and TCR clonality. In both murine tumor models used in our studies, the levels of tumor MHC Class I (~ 90%) and PD-L1 (Fig. 1a) in vivo are very similar within each treatment cohort, as is their capacity to mount an IFNγ response (Figs. 6 and 7). Future preclinical studies will examine other potential biomarkers of response, including TCR clonality.

An important reason for the combination of N-803 and αPD-L1 in these studies was to reduce immunosuppression in the TME. This is especially relevant as we demonstrate for the first time that N-803 induces upregulation of PD-L1 on immune cells in the primary tumor, lung, and spleen. MDSC, which are major contributors to immune suppression in the TME [39, 40], displayed an especially robust increase of PD-L1 expression. PD-L1 expression has been shown to correlate with MDSC immunosuppressive function [39–41], so it is possible that part of the enhanced anti-tumor efficacy observed with combination therapy is due to blocking PD-L1 on MDSC populations. We also observed significant reductions in G-MDSC/granulocyte populations in the lung with combination therapy. Investigating MDSC phenotype and function is of future interest.

Interestingly, while N-803 + αPD-L1 combination did significantly reduce 4T1 lung metastasis, the combination was unable to control primary tumor growth. Given the differences observed in the immune compartments in the primary tumor, lung, and spleen, which have never before been fully interrogated and compared, we hypothesize that the mechanism by which N-803 + αPD-L1 therapy alters lung tumor burden primarily occurs outside of the regulation of primary tumor growth. While there were minimal changes to immune cell infiltration and phenotype in the primary tumor, N-803 + αPD-L1 combination greatly enhanced immune cell numbers and activation in other compartments, including the lung vasculature and spleen. Thus, instead of preventing the initiation of the metastatic process, it is possible that N-803 + αPD-L1 treatment promotes NK and CD8^+^ T cell killing of metastasizing tumor cells. This is supported by reports showing that NK cells can mediate tumor surveillance and control through lysis of tumor cells at peripheral sites, including in the vasculature [42–44]. Furthermore, NK cells have been shown to be essential for the regulation of metastasis by promoting tumor cell killing during the epithelial to mesenchymal transition without affecting primary tumor growth [45], and NK cells have also been visualized at the invasive margin of NSCLC tumors in patients [46]. Given that NK cells were 60% of total immune cells in the vasculature after treatment with either N-803 or N-803 + αPD-L1, this result may explain why NK cells were more necessary for protection against lung metastasis versus CD8^+^ T cells. However, this increase in NK cells alone by N-803 was not solely responsible for the anti-tumor efficacy of N-803 + αPD-L1, as the addition of αPD-L1 further decreased lung tumor burden. We hypothesize that this additional efficacy is attributed to the effect that CD8^+^ T cells have on survival and growth of the metastases within the lung parenchyma, as N-803 + αPD-L1 specifically increased in CD8^+^ T cell function in this compartment versus all other treatments. Inducing these same phenotypic and functional changes on NK and CD8^+^ T cells within the primary TME could drastically decrease primary tumor burden. While we observed αPD-L1 monoclonal antibody localization in the primary TME (data not shown), whether N-803 also permeates into the primary tumor remains to be determined. Escorting N-803 to the TME by altering tumor permissiveness or by adding a tumor-targeting component such as αPD-L1 to N-803 may further improve anti-tumor efficacy through in-situ activation of NK and CD8^+^ T cells.

## Conclusions

For the first time, we demonstrate that the combination of N-803 + αPD-L1 therapy is well-tolerated and induces significant anti-tumor efficacy in multiple murine models of solid carcinomas that are non- and/or minimally responsive to either monotherapy. Furthermore, we evaluated the immune-associated effects of N-803 + αPD-L1 in multiple compartments, including the primary tumor, the lung as a site of metastasis, and spleen. We determined that this robust reduction in tumor burden is driven by NK and CD8^+^ T cells, which display an activated phenotype and greatly enhanced functionality upon N-803 + αPD-L1 treatment. These results provide the rationale for the clinical combination of N-803 with PD-L1-targeting agents for the treatment of both αPD-L1–responsive and αPD-L1–refractory tumors.

## Additional file

Additional file 1: **Table S1.** List of flow cytometry antibodies used for analysis of murine immune cell populations. **Table S2.** Flow cytometry gating strategy used for identification of murine immune cell populations. **Figure S1.** Validation of intravascular CD45-antibody labeling for the discrimination of vascular- versus parenchymal-resident immune cells in the spleen, lung, and primary tumor. **Figure S2.** Dose escalation of N-803 in combination with a clinical relevant dose of αPD-L1. **Figure S3.** Combination of N-803+αPD-L1 reduces MC38-CEA primary tumor burden and increases survival. **Figure S4.** Depletion efficiency of CD8 and NK cell depletions, and requirement of CD4^+^ T cells for anti-tumor efficacy of N-803 and αPD-L1. **Figure S5.** CD8^+^ T cells and NK cells are responsible for MC38-CEA anti-tumor efficacy. **Figure S6.** N-803+αPD-L1 combination decreases CD4^+^ T cell and Treg numbers in the lung vasculature. **Figure S7.** N-803+αPD-L1 combination reduces G-MDSC numbers in the lung vasculature. **Figure S8.** N-803 monotherapy and combination of N-803+αPD-L1 promotes an activated NK cell phenotype and increases NK function. **Figure S9.** CD8^+^ T cell expression of PD-1 is significantly reduced in lung parenchyma and vasculature after N-803+αPD-L1 treatment. **Figure S10.** Combination of N-803+αPD-L1 increases effector function of CD8^+^ T cells. (PDF 554 kb)

## Abbreviations

6- TG: 6-Thioguanine
CEA: Carcinoembryonic antigen
DLT: Dose limiting toxicity
DP: Double producing
G-MDSC: Granulocytic myeloid derived suppressor cell
gMFI: geometric mean fluorescence intensity
i.p.: Intraperitoneal
i.t.: Intratumoral
i.v.: Intravenous
IFNγ: Interferon gamma
IL: Interleukin
M-MDSC: Monocytic MDSC
mOS: median overall survival
NK: Natural killer
NSCLC: Non-small cell lung cancer
PBS: Phosphate buffered saline
PD-1: Programmed cell death-1
PD-L1: Programmed cell death ligand-1
r: Recombinant
s.c.: Subcutaneous
SP: Single producing
T_CM_: Central memory T cell
T_eff_: T cell effector
T_EM_: Effector memory T cell
TIL: Tumor-infiltrating lymphocyte
TME: Tumor microenvironment
TN: Triple negative
TNFα: Tumor necrosis factor alpha
T_reg_: Regulatory T cell
γc: Gamma c
μg: Microgram
μl: Microliter
