# Supplementary material for: Mechanisms involved in IL-15 superagonist enhancement of anti-PD-L1 therapy
Source: J Immunother Cancer. 2019 Mar 21;7:82. doi: 10.1186/s40425-019-0551-y (PMC6429734; doi:10.1186/s40425-019-0551-y)
Supplement: Supplementary file 1 — Table S1. List of flow cytometry antibodies used for analysis of murine immune cell populations. Table S2. Flow cytometry gating strategy used for identification of murine immune cell populations. Figure S1. Validation of intravascular CD45-antibody labeling for the discrimination of vascular- versus parenchymal-resident immune cells in the spleen, lung, and primary tumor. Figure S2. Dose escalation of N-803 in combination with a clinical relevant dose of αPD-L1. Figure S3. Combination of N-803+αPD-L1 reduces MC38-CEA primary tumor burden and increases survival. Figure S4. Depletion efficiency of CD8 and NK cell depletions, and requirement of CD4+ T cells for anti-tumor efficacy of N-803 and αPD-L1. Figure S5. CD8+ T cells and NK cells are responsible for MC38-CEA anti-tumor efficacy. Figure S6. N-803+αPD-L1 combination decreases CD4+ T cell and Treg numbers in the lung vasculature. Figure S7. N-803+αPD-L1 combination reduces G-MDSC numbers in the lung vasculature. Figure S8. N-803 monotherapy and combination of N-803+αPD-L1 promotes an activated NK cell phenotype and increases NK function. Figure S9. CD8+ T cell expression of PD-1 is significantly reduced in lung parenchyma and vasculature after N-803+αPD-L1 treatment. Figure S10. Combination of N-803+αPD-L1 increases effector function of CD8+ T cells. (PDF 554 kb) [file 40425_2019_551_MOESM1_ESM.pdf]

SUPPLEMENTARY FIGURES

Knudson, et al. Mechanisms involved in IL-15 superagonist enhancement of anti-PD-L1 therapy

Antibody	Clone	Company
FoxP3	R16-715	BD Biosciences
CD62L	MEL-14	BD Biosciences
CD44	IM7	BD Biosciences
CD3ε	2C11	BD Biosciences
CD247	MIH5	BD Biosciences
CD11b	M1/70	BD Biosciences
IFNγ	XMG1.2	BD Biosciences
TNFα	MPG-XT22	BD Biosciences
CD8α	53-6.7	ThermoFisher Scientific
Ki67	SolA15	ThermoFisher Scientific
FoxP3	FJK-16s	ThermoFisher Scientific
NKp46	29A1.4	ThermoFisher Scientific
NKG2D	CX5	ThermoFisher Scientific
CD279	J43	ThermoFisher Scientific
CD8β	53-5.8	Biolegend
Ly6C	HK1.4	Biolegend
Ly6G	1A8	Biolegend
CD49b	DX5	Biolegend
CD45.2	104	Biolegend
CD4	RM4-4	Biolegend
CD4	RM4-5	Biolegend
Granzyme B	GB11	Invitrogen

Table S1. List of flow cytometry antibodies used for analysis of murine immune cell populations.

**Cell Population**	**Flow Cytometry Gating Strategy**
$CD8^+$ T Cells	Live/$CD45.2^+$/$CD3\varepsilon^+$/$CD8\alpha^+$
$CD4^+$ T Cells	Live/$CD45.2^+$/$CD3\varepsilon^+$/$CD4^+$/$FoxP3^-$
$CD4^+$ T_{reg}	Live/$CD45.2^+$/$CD3\varepsilon^+$/$CD4^+$/$FoxP3^+$
NK Cells	Live/$CD45.2^+$/$CD3\varepsilon^-$/$CD49b^+$
G-MDSC/Granulocytes	Live/$CD45.2^+$/$CD11b^+$/$Ly6C^{lo}$/$Ly6G^{hi}$
M-MDSC/Monocytes	Live/$CD45.2^+$/$CD11b^+$/$Ly6C^{hi}$/$Ly6G^{lo}$
$CD45^+$ Cells	Live/$CD45.2^+$
$CD45^-$ Cells	Live/$CD45.2^-$

Table S2. Flow cytometry gating strategy used for identification of murine immune cell populations.

Figure S1

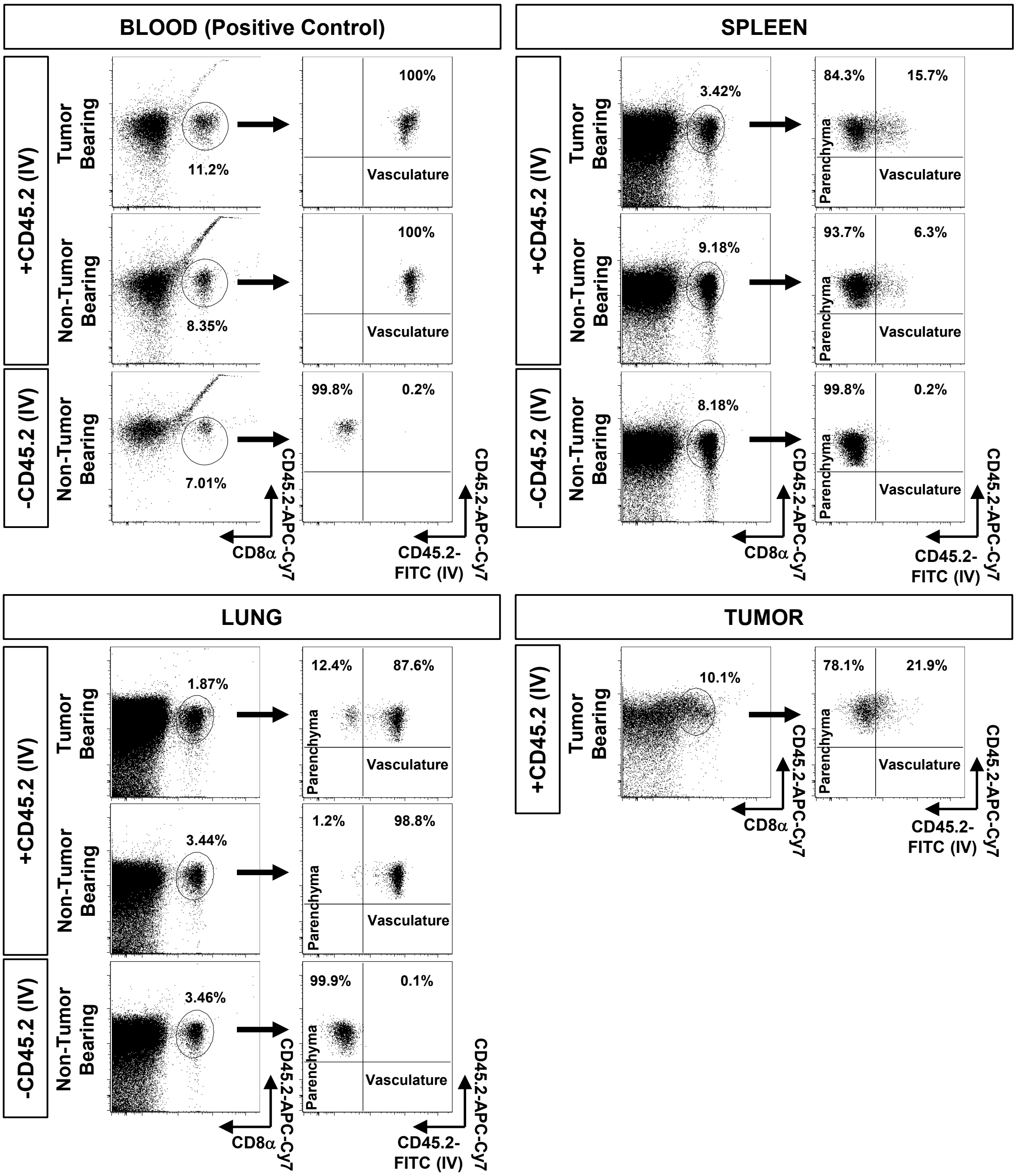

Figure S1. Validation of intravascular CD45-antibody labeling for the discrimination of vascular- versus parenchymal-resident immune cells in the spleen, lung, and primary tumor. 4T1 tumor implant was performed as in Figure 1. Intravascular labeling of all hematopoietic cells using an anti-CD45.2-FITC antibody was performed. Dot plots show examples of $CD8^+$ T cell populations in the blood (positive control), spleen, lung, and tumor from i.v.-labeled 4T1 tumor-bearing mice and i.v.-labeled and i.v.–non-labeled naïve (non-tumor bearing) control mice after subsequent staining with anti-CD45.2-APC-Cy7 and immune related markers. Lung parenchyma-resident immune cells were identified as CD45.2-APC-$Cy7^+$/CD45.2-$FITC^-$ and lung vascular-resident immune cells were identified as CD45.2-APC-$Cy7^+$/CD45.2-$FITC^+$. Data representative of 5 independent experiments, n=4-5 mice/group per experiment.

Figure S2

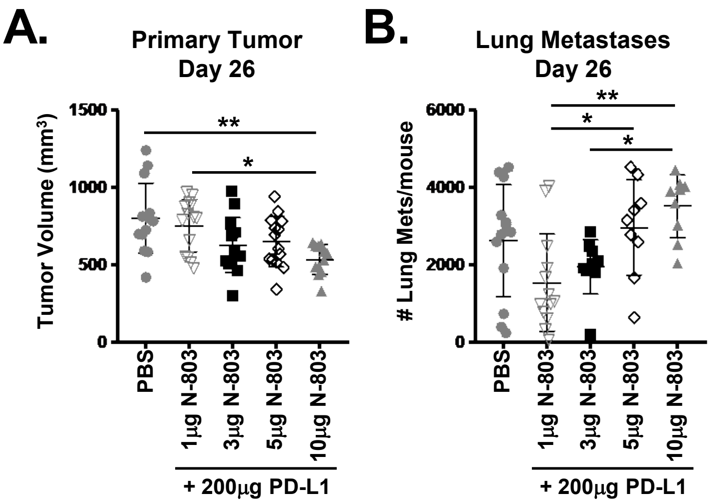

C.

Lung Metastases

# Lung Mets	PBS (n=13)	1µg N-803+ 200µg PD-L1 (n=13)	3µg N-803+ 200µg PD-L1 (n=10)	5µg N-803+ 200µg PD-L1 (n=9)	10µg N-803+ 200µg PD-L1 (n=9)
≥ 4000	3/13 (23%)	1/13 (8%)	0/10 (0%)	2/9 (22%)	3/9 (33%)
≤ 4000	10/13 (77%)	12/13 (92%)	0/10 (0%)	7/9 (78%)	6/9 (67%)
≤ 3000	7/13 (54%)	11/13 (85%)	10/10 (100%)	4/9 (44%)	2/9 (22%)
**≤ 2000**	**4/13 (31%)**	**10/13 (77%)**	**5/10 (50%)**	**2/9 (22%)**	**0/9 (0%)**
**≤ 1000**	**3/13 (23%)**	**6/13 (46%)**	**1/10 (10%)**	**1/9 (11%)**	**0/9 (0%)**
≤ 500	2/13 (15%)	2/13 (15%)	1/10 (10%)	0/9 (0%)	0/9 (0%)
≤ 100	1/13 (8%)	1/13 (8%)	0/10 (0%)	0/9 (0%)	0/9 (0%)
0	0/13 (0%)	0/13 (0%)	0/10 (0%)	0/9 (0%)	0/9 (0%)
Mean	2630	1545	1949	2972	3520
% Reduction		41%	26%	-13%	-33%

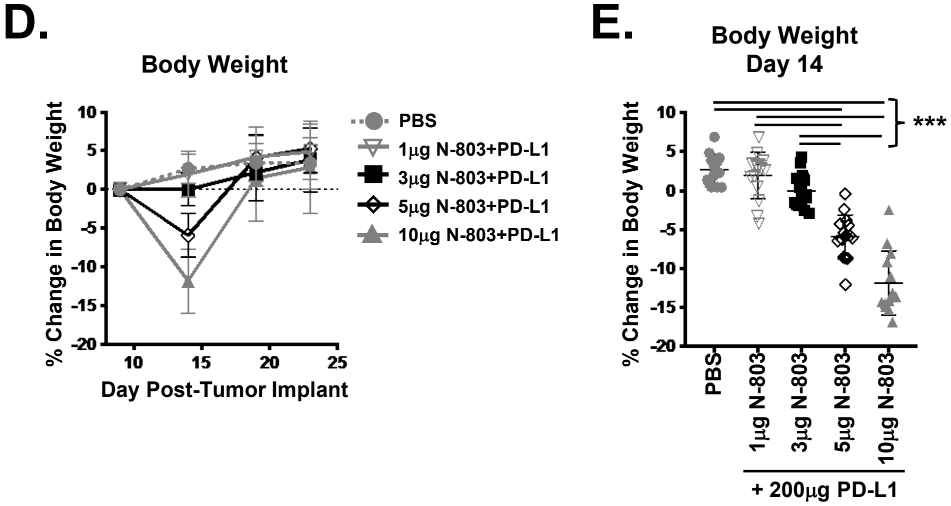

Figure S2. Dose escalation of N-803 in combination with a clinical relevant dose of αPD-L1. Mice were implanted with 4T1 tumors as in Figure 1 and treated at days 9 and 13 with 1µg, 3µg, 5µg, or 10µg N-803 (s.c) and 200µg αPD-L1 (i.p.) on days 9, 11, and 13. Graphs show tumor volumes of individual mice **(A)** or number of lung metastases in individual mice at day 26 post-tumor implant **(B)** as mean±SD. **(C)** Table shows distribution of lung metastases per mouse. **(D-E)** Graphs show % reduction in body weight (versus day 9 pre-treatment) over the course of the experiment **(D)** or of individual mice at day 14 (1 day post-final treatment) **(E)**. Data from 1 experiment, n=9-13 mice.

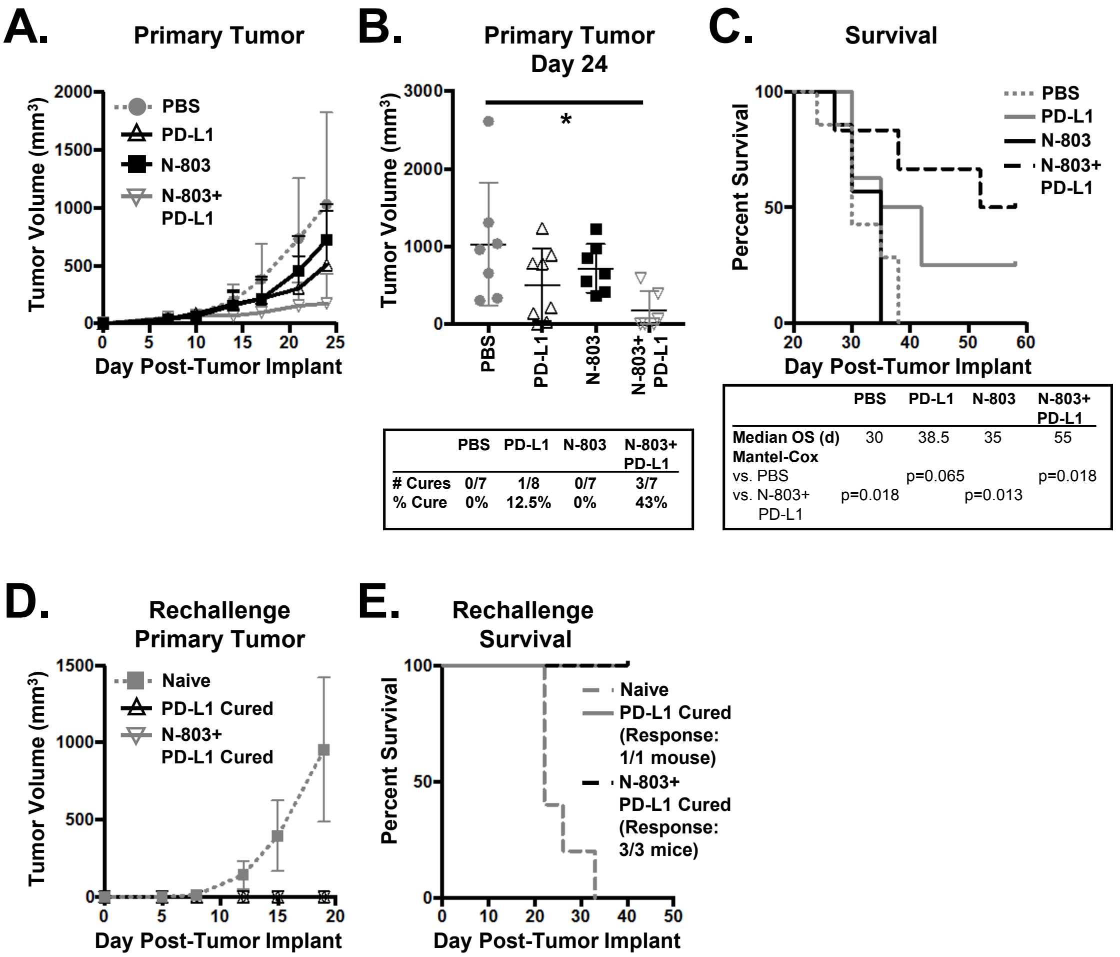

	PBS	PD-L1	N-803	N-803+ PD-L1
# Cures	0/7	1/8	0/7	3/7
% Cure	0%	12.5%	0%	43%

	PBS	PD-L1	N-803	N-803+ PD-L1
Median OS (d)	30	38.5	35	55
Mantel-Cox				
vs. PBS		p=0.065		p=0.018
vs. N-803+ PD-L1	p=0.018		p=0.013	

Figure S3. Combination of N-803+αPD-L1 reduces MC38-CEA primary tumor burden and increases survival. (A-C) 5x10[5] MC38-CEA tumor cells were implanted into the flank of female C57BL/6-CEA mice. When tumor volumes reached ~50mm^3, mice were treated at days 7 and 11 with 1μg N-803 (s.c.) and/or 200μg αPD-L1 (i.p.) on days 7, 9, and 11. Primary tumor growth curves **(A)** and tumor volumes of individual mice at day 24 (inset: % cured mice) **(B)** show mean±SD. **(C)** Survival curves (inset: mOS) show % survival. **(D-E)** At least 1 month after tumor cure (day 55), cured mice and paired naïve C57BL/6-CEA were implanted with 5x10[5] MC38-CEA tumor cells. **(D)** Primary tumor growth curves show mean±SD. **(E)** Survival curves (inset: # mice with memory response) show % survival. Data are from 1 independent experiment, n=7-8 mice.

Figure S4

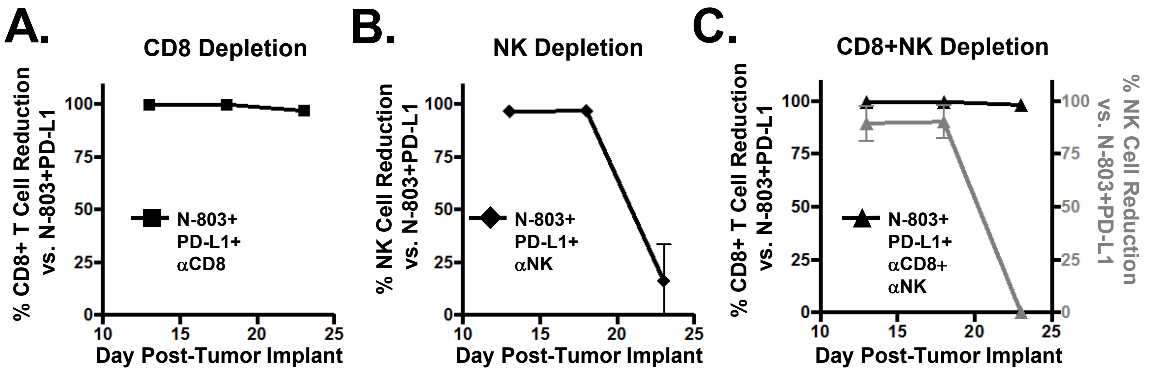

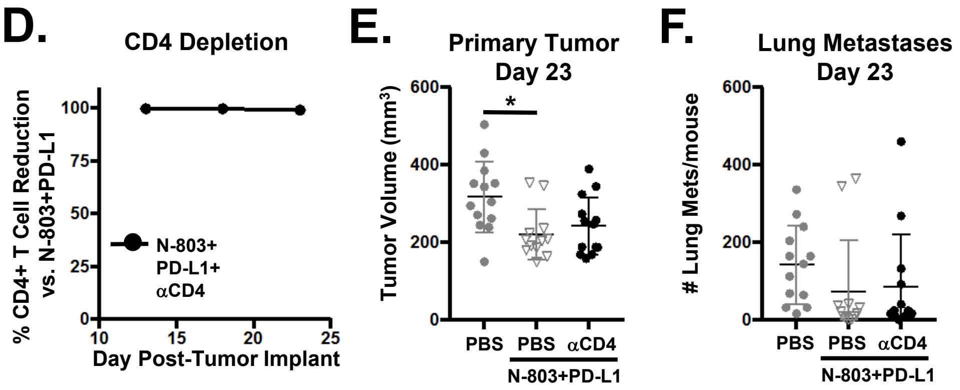

G. **Lung Metastases**

# Lung Mets/ mouse	PBS (n=13)	N-803+ PD-L1 (n=12)	N-803+ PD-L1+αCD4 (n=13)
≤ 500	13/13 (100%)	12/12 (100%)	13/13 (100%)
≤ 200	9/13 (69.2%)	10/12 (83.3%)	11/13 (84.6%)
≤ 100	3/13 (23%)	10/12 (83.3%)	10/13 (76.9%)
**≤ 50**	**3/13 (23%)**	**10/12 (83.3%)**	**9/13 (69.2%)**
≤ 10	0/13 (0%)	5/12 (41.7%)	2/13 (15.4%)
0	0/13 (0%)	2/12 (16.7%)	1/13 (7.6%)
Mean	142.2	72.7	85.3
% Reduction	0%	49%	40%

Figure S4. (A-C) Depletion efficiency of CD8 and NK cell depletions. Mice were implanted with 4T1 tumors as in Figure 1 and treated at days 13 and 17 with N-803 and αPD-L1 on days 13, 15, and 17. CD8-expressing cells and NK cells were depleted on days 10, 11, 12, 16, and 19 using 100μg anti-CD8 and/or 25μl anti-asialo-GM1 (i.p.). Depletion efficiency of $CD8^+$ T cells **(A)**, NK cells **(B)**, or $CD8^+$ T and NK cells **(C)** was examined in the blood on days 13, 18, and 23 by flow cytometry. Data combined from 2 independent experiments, n=3 mice/group per experiment. **(D-G) $CD4^+$ T cells do not contribute to the anti-tumor efficacy of N-803+αPD-L1 combination.** Mice were implanted with 4T1 tumors as in Figure 1 and treated at days 13 and 17 with N-803 and αPD-L1 on days 13, 15, and 17. CD4-expressing cells were depleted on days 10, 11, 12, 16, and 19 using 100μg anti-CD4. **(D)** CD4 depletion efficiency in the blood of 3 mice per group was determined on days 13, 18, and 23 by flow cytometry. Graphs show tumor volumes of individual mice **(E)** or number of lung metastases in individual mice at day 23 post-tumor implant **(F)** as mean±SD. **(G)** Table shows distribution of lung metastases per mouse. Data from 1 experiment, n=11-13 mice.

Figure S5

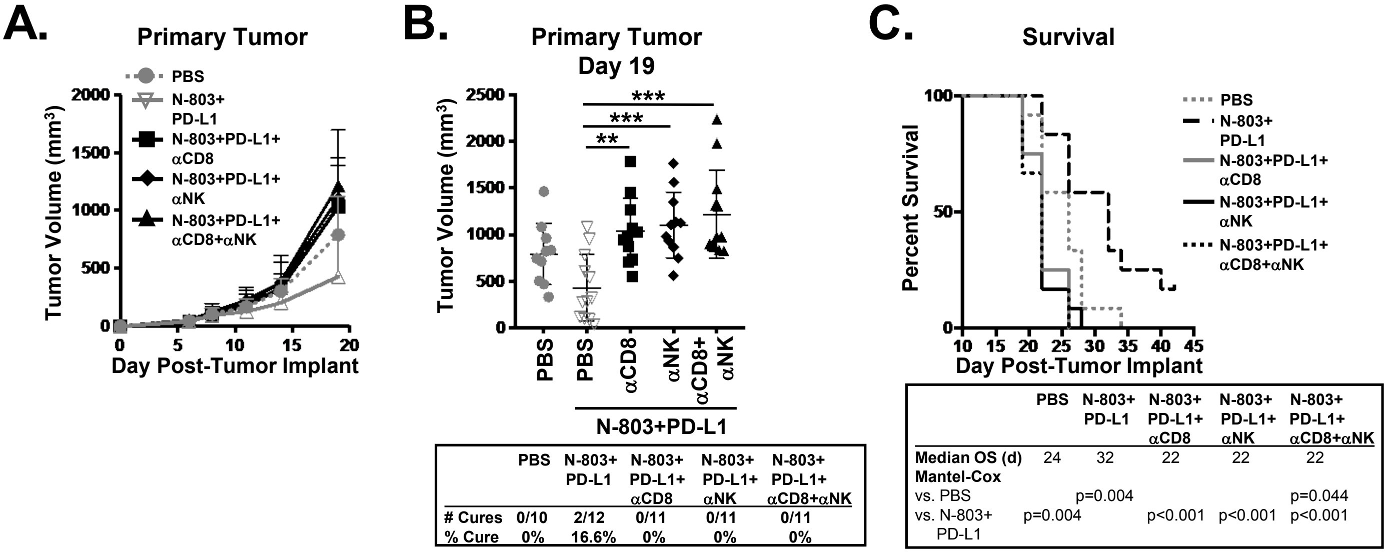

	PBS	N-803+ PD-L1	N-803+ PD-L1+ αCD8	N-803+ PD-L1+ αNK	N-803+ PD-L1+ αCD8+αNK
# Cures	0/10	2/12	0/11	0/11	0/11
% Cure	0%	16.6%	0%	0%	0%

	PBS	N-803+ PD-L1	N-803+ PD-L1+ αCD8	N-803+ PD-L1+ αNK	N-803+ PD-L1+ αCD8+αNK
Median OS (d)	24	32	22	22	22
Mantel-Cox					
vs. PBS		p=0.004			p=0.044
vs. N-803+ PD-L1	p=0.004		p<0.001	p<0.001	p<0.001

Figure S5. CD8⁺ T cells and NK cells are responsible for MC38-CEA anti-tumor efficacy. $3x10^5$ MC38-CEA tumor cells were implanted into the flank of female C57BL/6-CEA mice. When tumor volumes reached ~50mm^3, mice were treated at days 8 and 12 with 1μg N-803 (s.c.) and/or 200μg αPD-L1 (i.p.) on days 8, 10, and 12. CD8-expressing cells and NK cells were depleted on days 6, 7, 8, 11, and 14 using 100μg anti-CD8 and/or 25μl anti-asialo-GM1 (i.p.). Primary tumor growth curves **(A)** and primary tumor volumes of individual mice at day 19 (inset: % cured mice) **(B)** show mean±SD. **(C)** Survival curves (inset: mOS) show % survival. Data are from 1 independent experiment, n=10-12 mice.

Figure S6

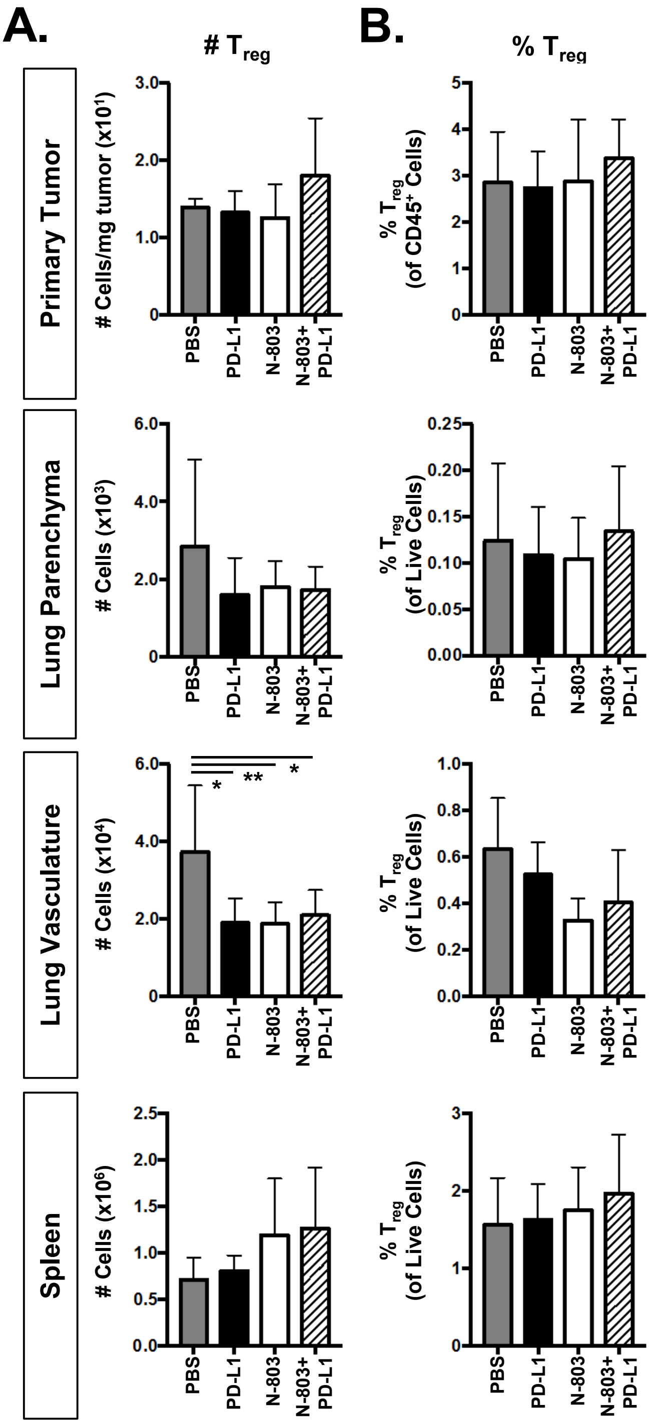

Figure S6. N-803+αPD-L1 combination decreases CD4$^+$ T cell and T_{reg} numbers in the lung vasculature. Mice were implanted with 4T1 tumors as in Figure 1 and treated at days 9 and 13 with N-803 and/or αPD-L1 on days 9, 11, and 13. Graphs show CD4$^+$ T_{reg} number (**A**) and frequency **(B)** in the primary tumor, lung parenchyma and vasculature, and spleen 24 hours after the last treatment (mean±SD). Data combined from 2 independent experiments, n=5 mice/group per experiment.

Figure S7

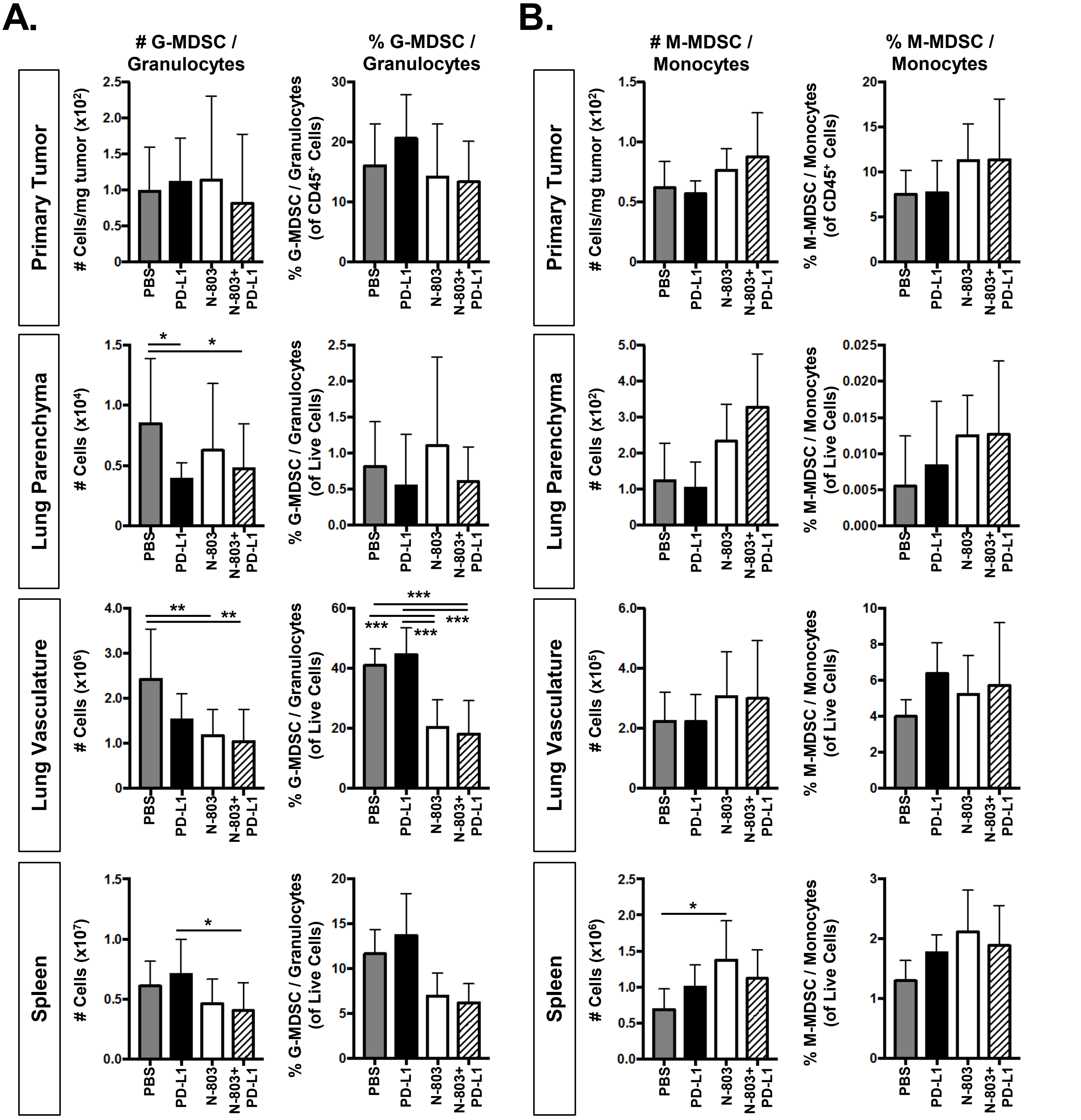

Figure S7. N-803+αPD-L1 combination reduces G-MDSC numbers in the lung vasculature. Mice were implanted with 4T1 tumors as in Figure 1 and treated at days 9 and 13 with N-803 and/or αPD-L1 on days 9, 11, and 13. Graphs show G-MDSC/Granulocyte **(A)** or M-MDSC/Monocyte **(B)** cell number (left panels) and frequency (right panels) in the primary tumor, lung parenchyma and vasculature, and spleen 24 hours after last treatment (mean±SD). Data combined from 2 independent experiments, n=5 mice/group per experiment.

Figure S8

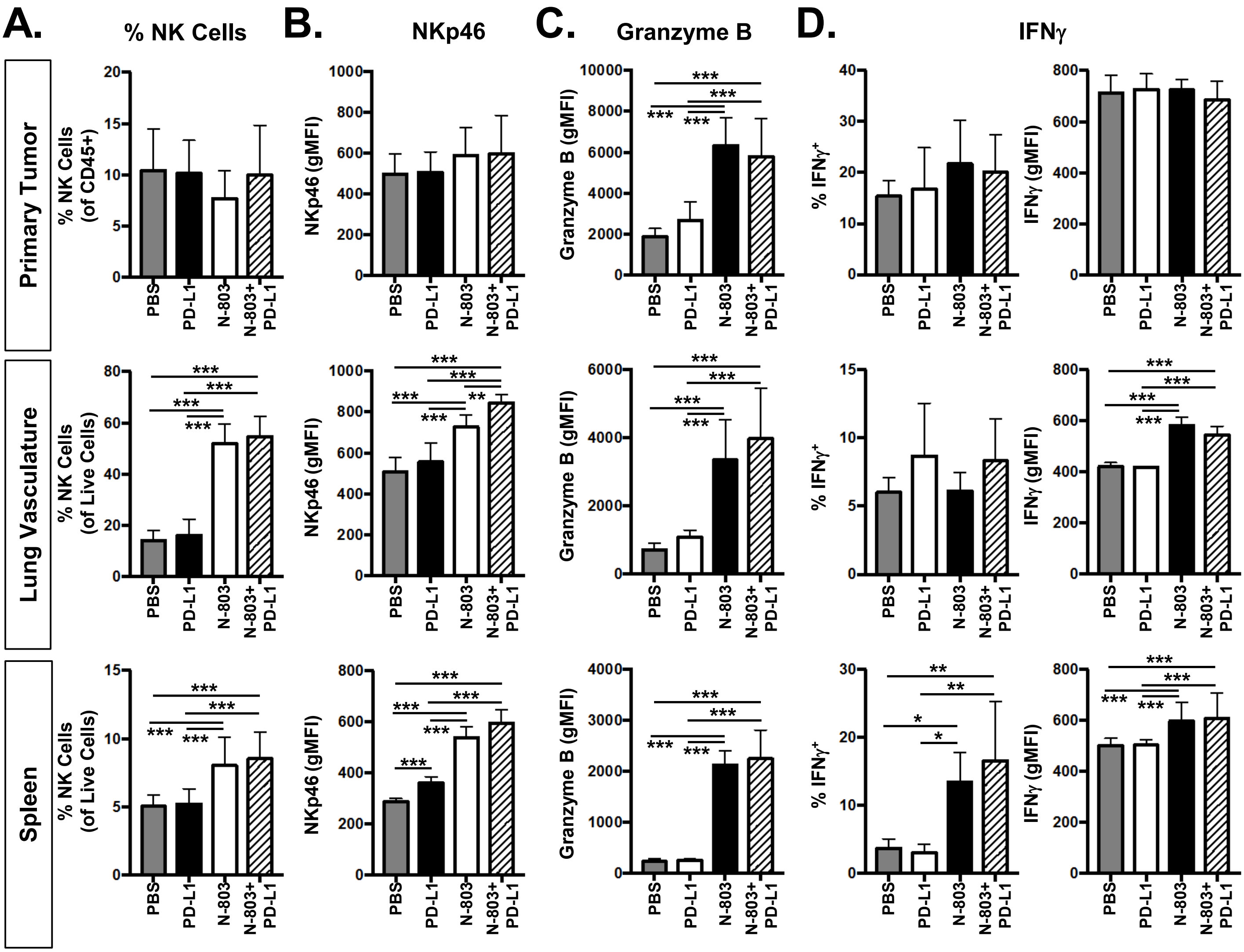

Figure S8. N-803 monotherapy and combination of N-803+αPD-L1 promotes an activated NK cell phenotype and increases NK function. Mice were implanted with 4T1 tumors as in Figure 1 and treated at days 9 and 13 with N-803 and/or αPD-L1 on days 9, 11, and 13. **(A-C)** NK cells were examined by flow cytometry in the primary tumor, lung vasculature, and spleen 24 hours after the last treatment. Graphs show NK cell frequency **(A),** expression of NKp46 (gMFI) in $NKp46^{+}$ NK cells **(B)**, and expression of Granzyme B (gMFI) in NK cells **(C)**. **(D)** Immune cells were stimulated with 50ng/ml PMA+500ng/ml ionomycin for 4 hours. Graphs show frequency of $IFN\gamma^{+}$ NK cells (left panel) and production of IFNγ (gMFI) (right panel) by NK cells. All graphs show mean±SD. Data combined from 2 independent experiments, n=5 mice/group per experiment.

Figure S9

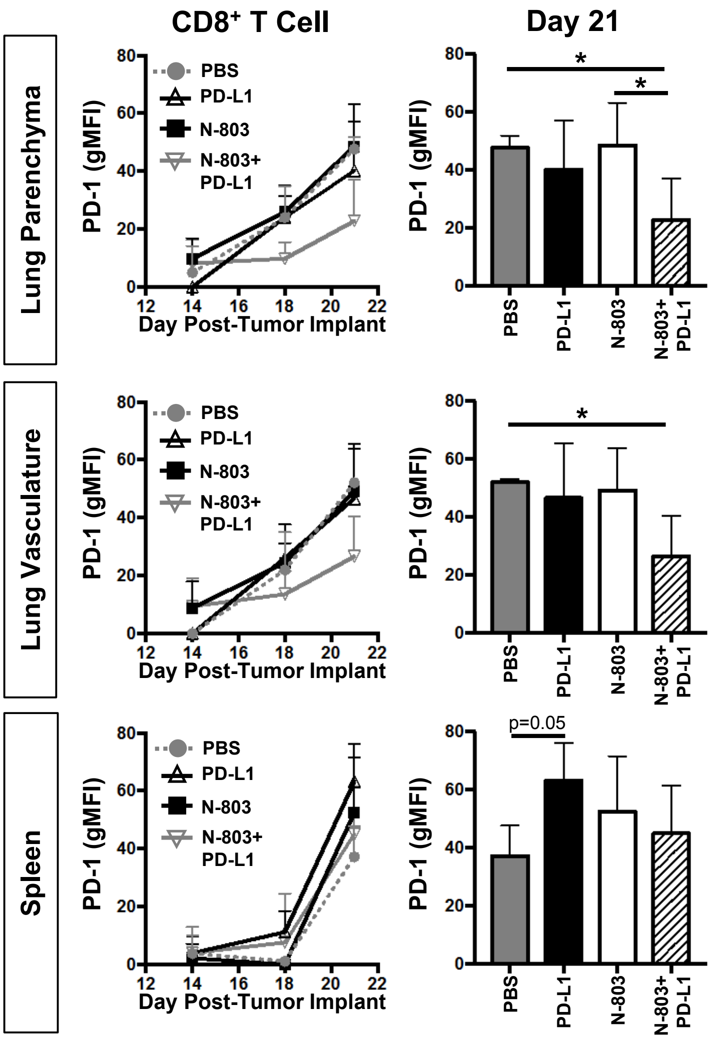

Figure S9. CD8$^+$ T cell expression of PD-1 is significantly reduced in lung parenchyma and vasculature after N-803+αPD-L1 treatment. Mice were implanted with 4T1 tumors as in Figure 1 and treated at days 9 and 13 with N-803 and/or αPD-L1 on days 9, 11, and 13. The primary tumor was resected at day 15. CD44^{hi} CD8$^+$ T cells were examined by flow cytometry for PD-1 expression in the lung parenchyma and vasculature and spleen at days 14, 18, and 21. Graphs of PD-1 expression (gMFI) of CD44^{hi} CD8$^+$ T cells at days 14, 18, and 21 (left panels) or day 21 only (right panels) show mean±SD. Data are from 1 experiment, n=5 mice.

Figure S10

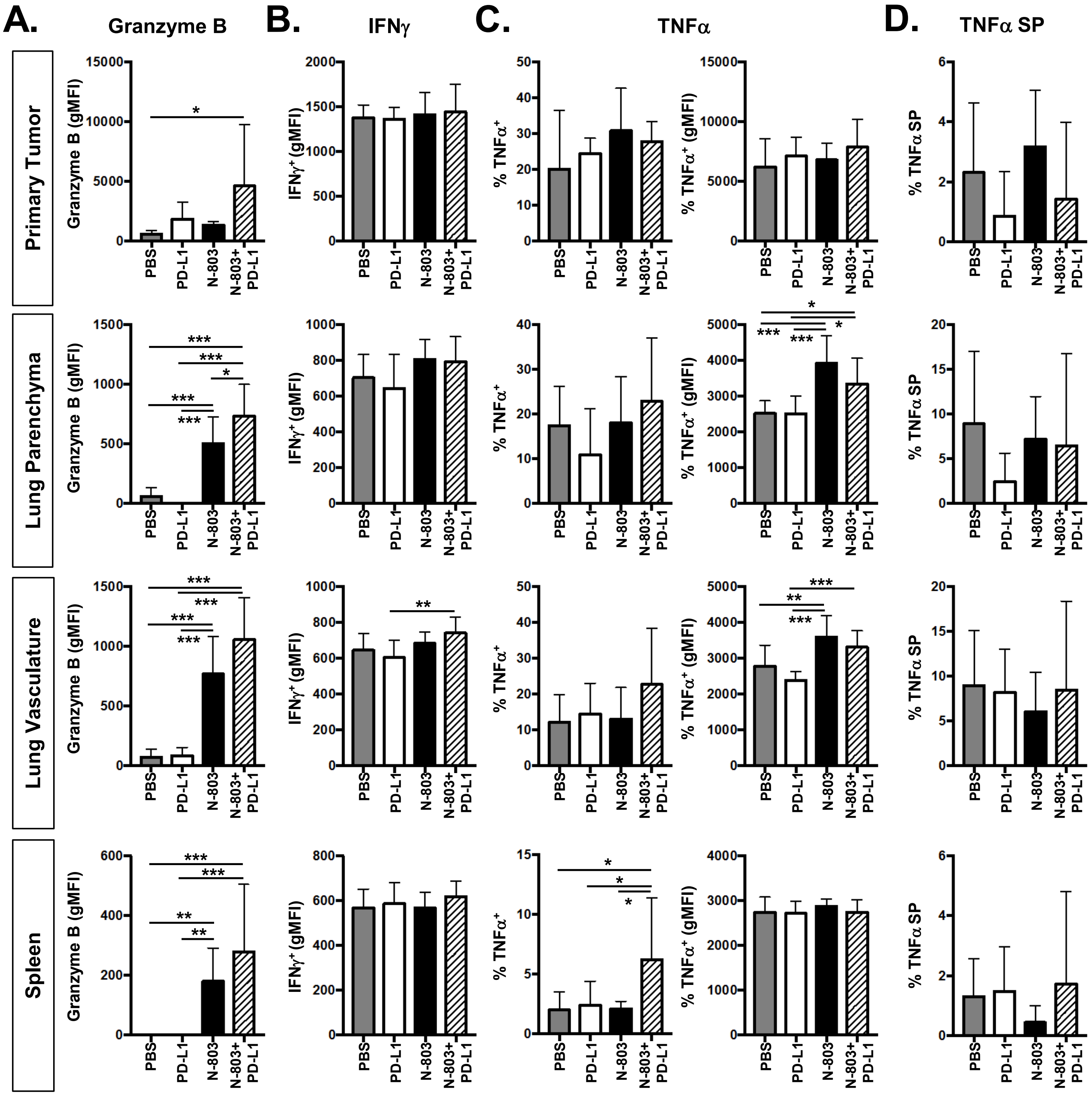

Figure S10. Combination of N-803+αPD-L1 increases effector function of CD8⁺ T cells. Mice were implanted with 4T1 tumors as in Figure 1 and treated at days 12 and 16 with N-803 and/or αPD-L1 on days 12, 14, and 16. CD8⁺ T cell effector cytokine and molecule production were examined by flow cytometry in the primary tumor, lung parenchyma and vasculature, and spleen 24 hours after last treatment. **(A)** Graphs show production of Granzyme B (gMFI) by CD44^{hi} CD8⁺ T cells. **(B-D)** Immune cells were stimulated with 1μg/ml αCD3+1μg/ml αCD28 for 4 hours. Graphs show production of IFNγ (gMFI) by CD44^{hi} CD8⁺ T cells **(B)**, frequency of TNFα⁺ CD44^{hi} CD8⁺ T cells **(C, left panel)** or production of TNFα (gMFI) by CD44^{hi} CD8⁺ T cells **(C, right panel)**, or frequency of TNFα-single producing (SP) CD44^{hi} CD8⁺ T cells **(D)**. All graphs show mean±SD. Data combined from 2 independent experiments, n=5 mice/group per experiment.
